# Origin of African *Physacanthus* (Acanthaceae) via Wide Hybridization

**DOI:** 10.1371/journal.pone.0055677

**Published:** 2013-01-30

**Authors:** Erin A. Tripp, Siti Fatimah, Iain Darbyshire, Lucinda A. McDade

**Affiliations:** 1 Rancho Santa Ana Botanic Garden, Claremont, California, United States of America; 2 University of Colorado at Boulder and University of Colorado Museum of Natural History, Boulder, Colorado, United States of America; 3 Royal Botanic Gardens, Kew, Richmond, Surrey, United Kingdom; University of Oxford, United Kingdom

## Abstract

Gene flow between closely related species is a frequent phenomenon that is known to play important roles in organismal evolution. Less clear, however, is the importance of hybridization between distant relatives. We present molecular and morphological evidence that support origin of the plant genus *Physacanthus* via “wide hybridization” between members of two distantly related lineages in the large family Acanthaceae. These two lineages are well characterized by very different morphologies yet, remarkably, *Physacanthus* shares features of both. Chloroplast sequences from six loci indicate that all three species of *Physacanthus* contain haplotypes from both lineages, suggesting that heteroplasmy likely predated speciation in the genus. Although heteroplasmy is thought to be unstable and thus transient, multiple haplotypes have been maintained through time in *Physacanthus*. The most likely scenario to explain these data is that *Physacanthus* originated via an ancient hybridization event that involved phylogenetically distant parents. This wide hybridization has resulted in the establishment of an independently evolving clade of flowering plants.

## Introduction

Although opinions regarding the importance of hybridization in biology have varied through time [1], [2], modern consensus leaves little doubt about its importance in evolutionary processes. For example, empirical studies in diverse groups including viruses, bacteria, plants, and animals have demonstrated higher fitness of hybrids relative to parents [3]. Evidence from plants [4], [5], animals [6], [7], and to a lesser degree fungi [8] demonstrates that hybridization can introduce novel, favorable genetic variation into populations, expedite ecological divergence, and contribute to the formation of new species. From a theoretical perspective, hybridization may facilitate saltation of populations across adaptive landscapes (e.g., from one adaptive peak or slope to another), thus permitting exploration of new fitness space without passage through maladaptive valleys [9].

The vast majority of examples of plant and animal hybridization involve closely related organisms [10], [11]. Studies from both classic [5], [12] and non-model [13] systems most frequently document hybridization near the species level. Less clear is the extent to which natural hybridization between evolutionarily distant taxa has played macroevolutionary roles that lead to independently evolving lineages. Recent rejection of the long-standing “Sax hypothesis” for origin of the apple subfamily via “wide hybridization” (in favor of hybrid ancestry involving close relatives) calls into question the importance of wide hybrids in plant evolution [14]. Indeed, it is axiomatic that hybridization capacity decreases over increasing evolutionary distance [15], [16]. Although there is a handful of examples of natural wide hybrids in plants [17], [18] and even fewer in animals [19], the vast majority of known wide hybrids are derived from artificial crosses, often for purposes of agronomic or horticultural improvement [20], [21]. However, progeny of both artificial and natural wide hybridization are usually sterile; in consequence, advanced generations are few and their long-term evolutionary significance is limited.

Remarkably, initial DNA sequence data from plants of the African genus *Physacanthus* suggested that (1) the genus is derived from wide hybridization between parents belonging to evolutionarily very distant lineages in the plant family Acanthaceae, a clade of >4,000 species, and that (2) long-term maintenance of heteroplasmy also characterizes the products of this wide hybridization. The latter may be as unexpected as the former. For some time, it has been known that ‘leakiness’ with respect to uniparental inheritance of organellar genomes can result in heteroplasmic individuals (i.e., those containing more than one organellar genome) [22] and heteroplasmy has been documented in diverse plant lineages [22], [23]. However, because heteroplasmy is considered to be unstable physiologically, it is believed to be transient [22], [23], but see [24]. We know of no studies that have documented long-term maintenance of heteroplasmy derived from distantly related parents.

We tested hypotheses of wide hybridization and heteroplasmy by reconstructing the phylogenetic placement of *Physacanthus* in the broader context of all Acanthaceae and by comparing morphological features of *Physacanthus* to its putative parental lineages. We explicitly explore (1) an alternative hypothesis to wide hybridization (i.e., retention of ancestral polymorphisms) by cloning genic regions from multiple individuals of *Physacanthus* as well as of plants representing other genera of Acanthaceae, and (2) an alternative hypothesis to maintenance of heteroplasmy through evolutionary time by testing for intermolecular chloroplast recombination. Our results document a case of natural wide hybridization that has led to establishment of a stable heteroplasmic multi-species lineage of flowering plants.

## Results and Discussion

### Molecular Evidence for Wide Hybridization and Heteroplasmy

**Figure 1:**
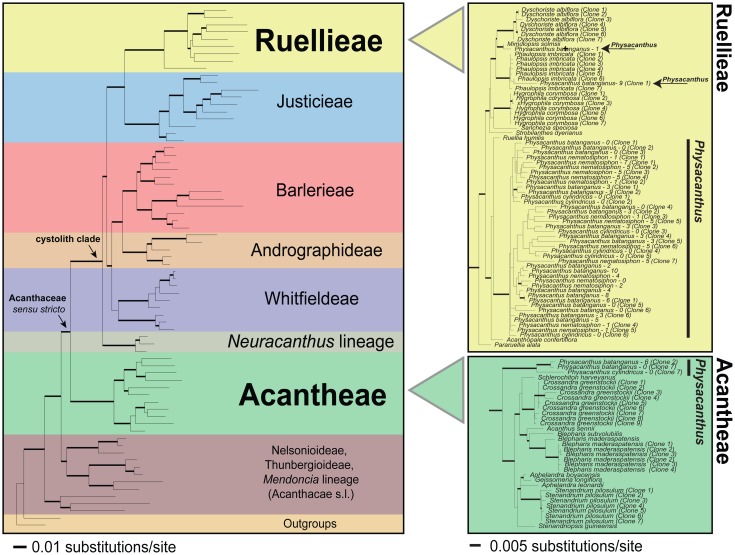
Concatenated seven-gene maximum likelihood analysis of Acanthaceae (>4,000 species) showing extensive molecular divergence between Acantheae and Ruellieae, the progenitor lineages of *Physacanthus* (left). Single chloroplast (*trnG-trnR*) gene tree showing *Physacanthus* sister to Acantheae as well as nested within Ruellieae (right). Thickened branches indicate ≥70% ML bootstrap.

**Figure 2:**
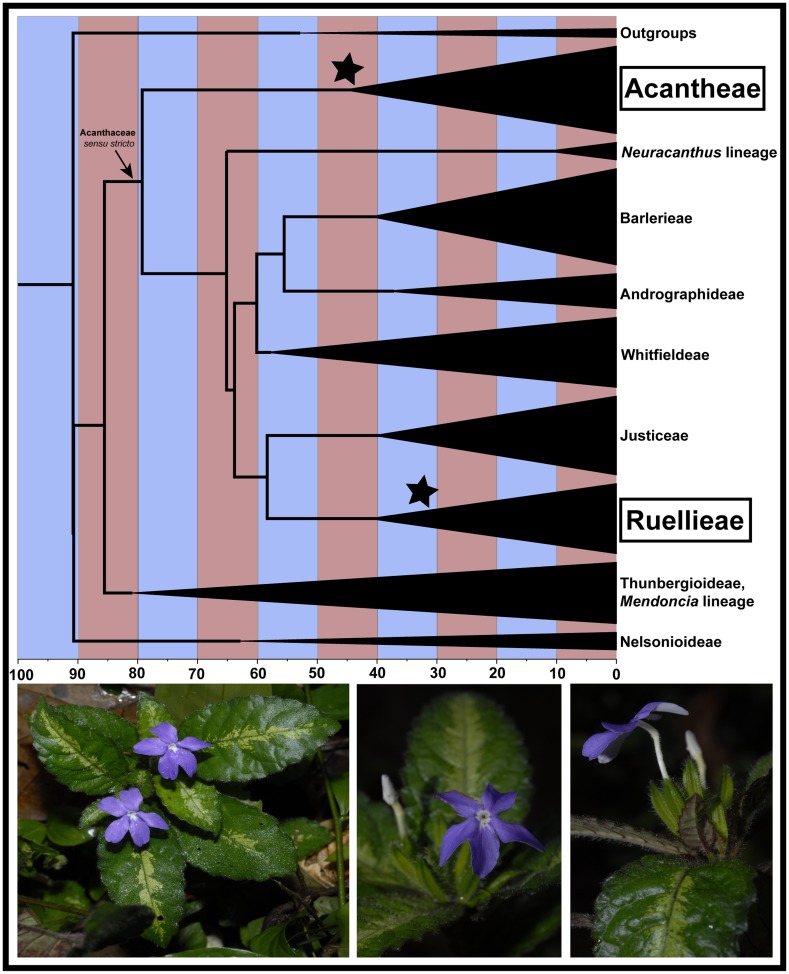
Time-calibrated phylogeny of Acanthaceae showing contemporaneous evolution of Acantheae (95% CI = 54–24 mya) and Ruellieae (95% CI = 55–31 mya). Stars indicate two internal fossil calibrations. All branches except placement of the *Neuracanthus* lineage are strongly supported. Photographs (by Martin Cheek) of living *Physacanthus batanganus* showing variegated leaves and unique morphology (see text).

Molecular data presented here demonstrate affinity of *Physacanthus* to two distantly related tribes of Acanthaceae: Ruellieae and Acantheae (Figures 1, S1). Pairwise sequence divergence values herein calculated for extant Acantheae and Ruellieae ranged from 13 to 31% (nuclear sequence data) or 8–18% (chloroplast data). These divergence values approximate the upper end of total sequence divergence within the family (Table 1). We utilized 20 different DNA extractions (representing multiple individuals of all three species; Table S1) and sequence data from seven loci (one nuclear, six chloroplast) to reconstruct phylogenetic placement of *Physacanthus*. Three loci placed *Physacanthus* only among Acantheae, one placed *Physacanthus* only among Ruellieae, two placed *Physacanthus* among both Acantheae and Ruellieae, and one placed *Physacanthus* among both Ruellieae and one unsupported branch proximal to Acantheae (placement sister to Acantheae could not be rejected; Text S1; Figures 1, S1; Table S2). These results were strongly supported by bootstrapping and were robust to extensive testing of alternative topological hypotheses (Table S2). Our results further reveal both Ruellieae-like and Acantheae-like sequences in all accessions of *Physacanthus* for which we were able to obtain more than one sequence (i.e., 10 of 20; Table S3). Thus, sequence data are consistent with a hypothesis of wide hybridization for the origin of *Physacanthus* accompanied by the establishment and long-term maintenance of heteroplasmy. Using two Acanthaceae fossils, we estimated divergence times across the family to provide a temporal context for the wide hybridization of *Physacanthus*. Our analyses indicate that Acantheae and Ruellieae diverged at least 65 mya ago (Figure 2; mean = 79 mya, 95% credibility interval = 65–102 mya), with diversifications of crown clades starting around 44 mya (Acantheae; 95% CI = 54–42 mya) or 40 mya (Ruellieae; 95% CI = 55–31 mya).

**Table 1:** Table **1.** Range of pairwise sequence divergences between Acantheae and Ruellieae (Row 1) compared to total range of sequence divergence across Acanthaceae *sensu stricto* (Row 2) (values are uncorrected “p” distances).

	ITS	*trnLF*	*trnTL*	*rps16*	*trnGR*	*trnGS*	*psbA-trnH*
**Acantheae vs. Ruellieae**	13–31%	8–17%	13–18%	9–13%	9–18%	15–21%	9–18%
**All Acanthaceae**	2–38%	1–17%	0–23%	0–15%	2–18%	4–21%	2–18%

As an alternative hypothesis to that of hybridization, we explored the possibility of retention of ancestral polymorphisms to explain presence of both Acantheae-like and Ruellieae-like chloroplast sequences in *Physacanthus*. If mutations that predated the divergence of Acantheae and Ruellieae were still undergoing lineage sorting, we would expect to recover a similar pattern of multiple phylogenetic placements for related genera of Acanthaceae. We conducted extensive cloning of five of the seven loci for *Physacanthus* (Table S3) plus three genera each of Acantheae (*Crossandra*, *Blepharis*, *Stenandrium*) and Ruellieae (*Hygrophila*, *Dyschoriste*, *Phaulopsis*). To minimize sampling bias, cloning effort was uniform across taxa. From more than 235 clones (summed across taxa and loci), we detected only one chloroplast polymorphism (i.e., one variant clone of *Stenandrium* in one locus; Figure S1b) for any accession of any taxon other than *Physacanthus* (Text S1). These data suggest that ancestral polymorphisms are unlikely to account for the observed data. Moreover, population genetic theory predicts that incomplete lineage sorting is most pronounced over small evolutionary distances, over short time scales, and in populations with large effective sizes [25]. This led Maddison [26] to conclude that “long narrow (width measured by effective population size) trees are nearly immune to deep coalescence (problems) whereas short wide trees may show many genes with deep coalescence ‘problems’.” Given these data, as well as the considerable temporal and evolutionary divergence between Acantheae and Ruellieae (Figures 1, 2, Table 1), two lineages at opposite phylogenetic extremes of a family of 4,000+ species, the ancestral polymorphism hypothesis is unlikely.

**Figure 3:**
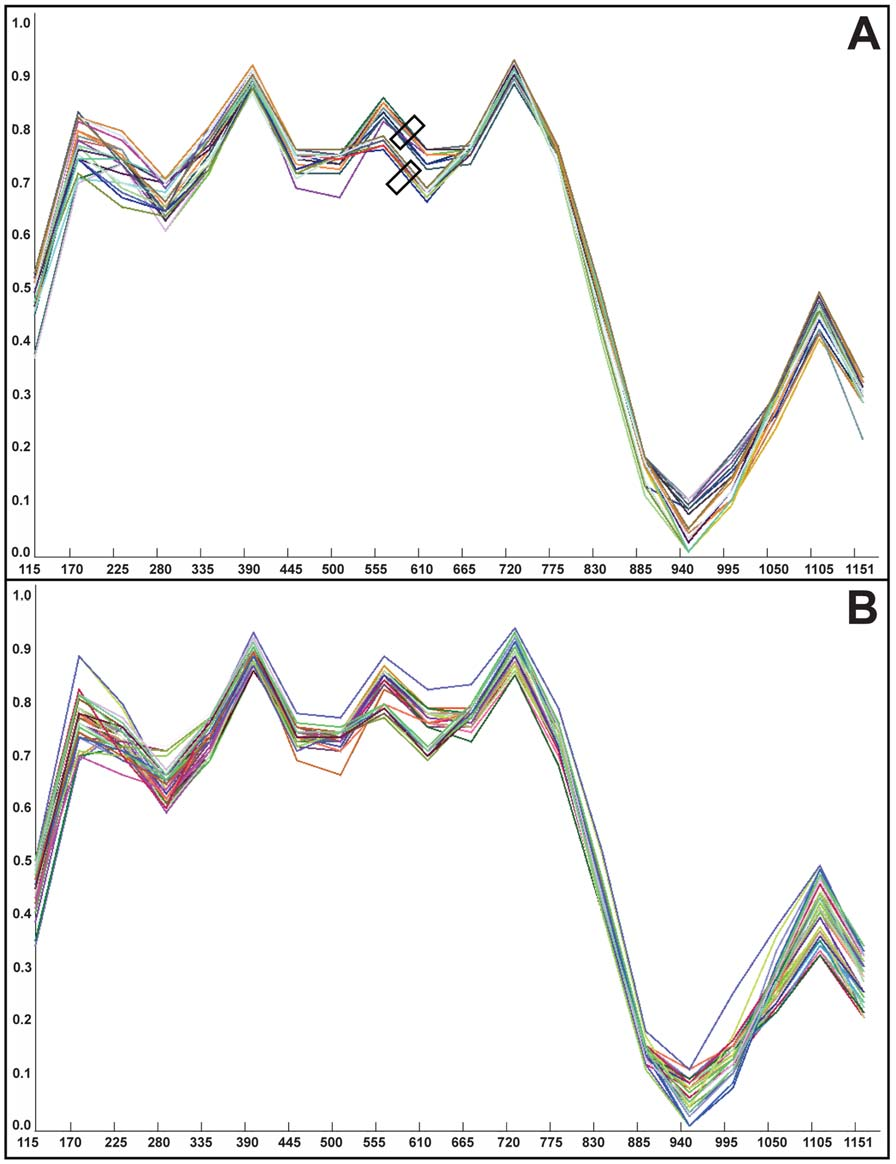
Two of numerous tests for intermolecular recombination among chloroplasts of Acantheae and Ruellieae as well as within Ruellieae and *Physacanthus* based on *trnG-trnR* data. In both figures, X-axis depicts aligned *trnG-trnR* sequence matrix ranging from 1 to 1151 base positions. Y-axis depicts percent sequence similarity. Figure A graphs all taxa of Acantheae (lower rectangle) and Ruellieae (upper rectangle) against that of *Physacanthus batanganus*-0 (Table S1). Figure B graphs all taxa of Acantheae, Ruellieae, and other *Physacanthus* against *Physacanthus-cylindricus*-0. Both *P. batanganus*-0 and *P. cylindricus*-0 are heteroplasmic (Figure 1). We detected no recombination. Both graphs based on sequence similarity thresholds beginning with <70% and jumping to >90% similarity (y axis), window sizes of 115 bp, and increment sizes of 55 bp (x axis).

As an alternative hypothesis to heteroplasmy to explain the observed data, we explored the possibility of intermolecular chloroplast recombination. We tested for recombination by using a sliding window sequence similarity approach in which a focal sequence is compared to sequences from the putative parents. Each of the 20 accessions of *Physacanthus* was individually treated as the focal sequence and compared against (1) all Acantheae and Ruellieae, (2) all Ruellieae only, and (3) all *Physacanthus* only. This three-tiered approach allowed us to test for recombination between the two parental lineages, within one of the two lineages (Ruellieae), and within *Physacanthus*. We rigorously explored varying thresholds of sequence similarity as well as sliding window and sampling interval sizes but detected no evidence for recombination (Figure 3). Absent data that would support a recombination event or events, a hypothesis of heteroplasmy is favored.

In flowering plants, uniparental (maternal) inheritance of chloroplasts is the rule such that products of hybridization should reveal the identity of the maternal parent only [23], although both paternal and biparental inheritance of organellar genomes are known from some groups. Previous work on three unrelated genera of Acanthaceae suggests that chloroplast inheritance is strictly maternal [27], [28]. Our finding of more than one sequence for chloroplast regions in all accessions of *Physacanthus* for which we were able to obtain sequence data from more than one locus (Table S3) and our failure to detect chloroplast recombination are consistent with the hypothesis that plants of this genus have both parental chloroplast haplotypes and that heteroplasmy has been maintained following leaky chloroplast inheritance in the genus. Notably, we have shown that heteroplasmy is not typical of Acanthaceae in that cloned sequences from six other taxa were homogeneous and were never placed in entirely different lineages, in contrast to those of *Physacanthus* (Figures 1, S1).

In contrast to ample evidence from chloroplast loci that suggests origin of *Physacanthus* via wide hybridization (Figures 1, S1a-S1e), nuclear DNA data failed to reveal evidence of both parental lineages. Only Acantheae-like sequences were obtained for nrITS+*5.8S* despite sequencing of as many as five clones per accession (Figure S1f). However, numerous studies have shown that the nrITS+*5.8S* region may rapidly homogenize to one or the other parental type independently of the rest of the nucleus [29], [30] such that our data in isolation do not constitute a strong test of a hypothesis of hybridization. Alternatively, it is possible that we did not sufficiently sequence clones ITS+*5.8S* to retrieve Ruellieae-like sequences. As organismal phenotypes are understood to be largely controlled by the nuclear and not organellar genomes [31], we here argue that the phenotype of *Physacanthus* itself provides considerable evidence of the contribution of both Acantheae and Ruellieae to the ancestry of *Physacanthus*.

### Morphological Evidence for Wide Hybridization and Heteroplasmy

**Figure 4:**
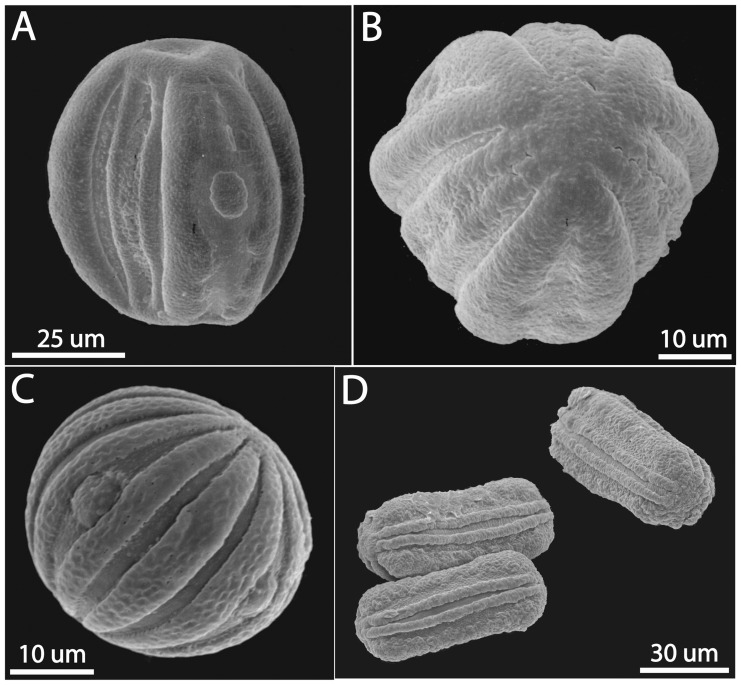
Pollen of *Physacanthus* has apertures consisting of a pore within a colpus, and has grooves between the apertures (A–B), just like pollen of Ruellieae (C). Pollen of Acantheae has apertures that consist only of a colpus and lack grooves between the apertures (D). A. *Physacanthus batanganus* (Bates 139, K). B. *Physacanthus batanganus* (Bates 139, K). C. *Hygrophila stricta* (Ruellieae; Meuer 10133, US). D. *Stenandriopsis guineensis* (Acantheae; Reitsma & Reitsma 705, RSA).

In remarkable concordance with molecular results discussed above, our study of morphological traits of *Physacanthus* (herein synthesized for the first time) confirms that these plants share features with both Acantheae and Ruellieae (Figures 4, S2, S3). As context for this finding, all phylogenetic studies to date have shown that Acantheae and Ruellieae are strongly supported lineages, are not sister tribes (Figure 1), and are each well marked by highly diagnostic morphological characters [32]–[34]. Interestingly, *Physacanthus* has been classified by taxonomists over the last century into either Acantheae or Ruellieae [35]–[37]. Uncertainty regarding the placement of *Physacanthus* can be understood by closer examination of morphology.

Like all plants of tribe Acantheae (ca. 500 species), plants of *Physacanthus* lack calcium oxalate leaf crystals (“cystoliths”; Figures 1, S2a) and have flowers with four stamens, each with a single celled anther. Conflicting with this are traits that argue instead for relationship of *Physacanthus* to the clade characterized by presence of leaf crystals that includes Ruellieae (i.e., pollen with compound germinal apertures; Figure 4), to Ruellieae + Justicieae (i.e., pollen with false apertures; Figure 4), or to Ruellieae (ca. 1200–1500 species) specifically (i.e., ovule number, twisted corolla form in bud; Figure S3). If Acantheae and Ruellieae were sister, placement of *Physacanthus* as the basal member of one or the other lineage might accommodate these patterns (together with adjustment of character evolution hypotheses). However, Acantheae and Ruellieae are not closely related (Figures 1, S1, Table 1). The finding that *Physacanthus* is morphologically an admixture between two lineages that are characterized by very different phenotypic traits (Figures 4, S2, S3) strongly supports our hypothesis of wide hybridization for the origin of *Physacanthus* (see Text S1 for additional discussion of morphology).

One further piece of phenotypic evidence lends additional support to the hypothesis of heteroplasmy: plants of *Physacanthus* have leaves that are frequently variegated (Figures 2, S3). Hybrid progeny derived from reciprocal crosses of two species of *Pelargonium* showed that biparental inheritance of chloroplasts was evident from plant phenotype: heteroplasmy was manifested in leaf variegation, with chlorotic portions possessing both parental haplotypes and green portions possessing just one of the two parental haplotypes [38]. Chlorotic portions of variegated leaves, like those of *Physacanthus*, may result from incompatibilities between nuclear and organellar genomes derived from the different parents, akin to Dobzhansky-Muller incompatibilities [39]. A qPCR-based method developed to quantify the degree of heteroplasmy in plants found significant variation in chloroplast number, identity, and location both within and among leaves, suggesting that the physical location of tissue sampled for DNA extraction may impact which haplotype is dominant in the sample [40]. We explored this phenomenon in *Physacanthus* by extracting DNA from three different museum specimens twice, each time using a different tissue sample. Two of the three re-extractions yielded evidence of different chloroplast haplotypes (Figures 1, S1a), which we interpret as evidence for heteroplasmy over evidence for contamination because (i) all sequences of *Physacanthus* were resolved consistently in only two of the seven major clades within the family and (ii) at the time of data generation for *Physacanthus* for this paper, there was no contemporaneous work in our lab on Acantheae. Thus, while re-extracting DNA from two of three specimens yielded different haplotypes, it did not suggest different hypotheses to explain the origin of *Physacanthus* other than that advanced here: wide hybridization between members of Acantheae and Ruellieae resulting in an evolutionarily stable heteroplasmic lineage.

Although parental contributors to this lineage of wide hybrid origin are unknown, our results suggest that one parent would likely have been of the stem lineage to the extant crown group of Acantheae. Within Acantheae, the basal rosettes and narrow floral tubes of *Physacanthus* are reminiscent of those of *Stenandriopsis* and *Crossandra*, which are early diverging within Acantheae. The second parent would have been a member of crown group Ruellieae. If extant, this parent was likely not from a group sampled in the present analysis (note that Ruellieae comprise >1,200 species in 48 genera of which only nine species in nine genera were sampled here). Clear candidates for parent of the hybrid are not apparent among extant West African Ruellieae. The inflated calyces of *Physacanthus* are reminiscent of *Satanocrater*, but species in the latter genus occupy very different habitats at present than do plants of *Physacanthus*.

### Maintenance of Heteroplasmy

It is important to note that we have documented heteroplasmy at the level of individuals. This pattern requires mechanisms both to establish heteroplasmy and to maintain it through multiple speciation events. The establishment of heteroplasmy must have involved chloroplast ‘leakage’ (i.e., transmission through pollen and eggs) during hybridization early in the history of the genus. Subsequent to this, maintenance of heteroplasmy requires a cellular mechanism to ensure transmission of a diversity of chloroplasts, specifically including both Acantheae- and Ruellieae-like organelles. Despite the notion that heteroplasmy is relatively unstable and thus a transient condition during organismal development and across generations [22], [23], it has apparently been maintained over evolutionary time in *Physacanthus*. One potential mechanism to explain heteroplasmic stability warrants further study: selection to maintain multiple haplotypes. Fitness consequences of heteroplasmy are expected to vary depending on molecular and environmental context. Severe heteroplasmic incompatibilities between cytoplasm and nuclear genomes can be lethal [39]. On the other hand, co-occurring chloroplast haplotypes that code for different gene products may positively affect plant growth and fitness over a plant's lifetime in a variable environment [41]. We propose that establishment of a viable and fertile phenotype in the early stages of the hybrid ancestry of *Physacanthus* benefited from–if not required–maintenance of chloroplasts from both parental lineages.

Five alternative hypotheses to that of long-term maintenance of heteroplasmy merit discussion. (1) Emigration of one parent's chloroplast genome to the nucleus [42] cannot be rejected, but we know of no instances in natural systems of entire organellar genomes (regions sequenced here span the chloroplast chromosome) being subsumed by the nucleus. (2) Horizontal gene transfer by a non-plant endosymbiont [43] or some other means [44] is not expected to produce the clear signal of hybridization in plant morphology that we have documented in *Physacanthus*. (3) Recombinant chloroplast molecules or fused chloroplasts have only rarely been documented [45], [46]. We detected no signature of recombination, and neither a recombinant nor a fused chloroplast molecule would have the capacity to produce segregating populations of chloroplast haplotypes during leaf development, assuming that leaf variegation in *Physacanthus* is a manifestation of such a process. (4) Mutation might help to explain conflicting chloroplast gene trees but we used non-coding sequences that are presumably not under strong selection and we would not expect random mutations to lead to strongly supported, contrasting phylogenetic signals. (5) Finally, artifactual results owing to PCR-induced recombination, *Taq* error, or laboratory contamination must be considered. If either PCR recombination or *Taq* error were issues, we would not expect consistent results involving the same parental lineages across all DNA extractions and loci. Rather, we would expect random distribution of the artifactual results. Moreover, there is ample signal from morphology for a hybrid origin of *Physacanthus*. Laboratory contamination is highly unlikely because when data were generated for this project there was no concurrent research that involved Acantheae. We intend to pursue future tests of these hypotheses via fieldwork to secure fresh plant materials that will permit gathering of more complete sequence data as well as additional sources of evidence (e.g., karyotype data).

Taken together, molecular and morphological data presented here are consistent with a hypothesis of origin of *Physacanthus* via wide hybridization involving distantly related parents. The fact that all three species of *Physacanthus* showed molecular evidence of the hypothesized wide hybridization event and that the three species in the genus are morphologically cohesive (i.e., they share the defining morphological feature of inflated calyces, Figures 2, S3) argues that the hybridization event occurred prior to speciation, giving rise to the common ancestor of the three species, Similar lines of evidence were used to argue that establishment of organellar heteroplasmy preceded species diversification in *Allium* [47]. The alternative hypothesis of re-establishing heteroplasmy every generation via hybridization seems extremely implausible (and would require that parental sources of both chloroplast lineages remain in sympatry), especially given that modern geographic ranges of the three species of *Physacanthus* are distinct. While we cannot reject this hypothesis, repeated establishment of heteroplasmy *as the sole means of explaining the data* is far less parsimonious and requires more elaborate scenarios than the wide hybridization plus maintenance of heteroplasmy hypothesis that we advance.

It is, however, possible, and perhaps likely, that some subsequent chloroplast introgression followed the initial wide hybridization event that established heteroplasmy. In the simplest scenario of a single ancient hybridization to establish heteroplasmy via leaky chloroplast inheritance, we would expect all accessions of *Physacanthus* to be monophyletic within both Acantheae and Ruellieae. Instead, accessions of *Physacanthus* were often not monophyletic *within* one of the two parental lineages—Ruellieae. The initial hybridization may have been followed by chloroplast introgression between *Physacanthus* and plants of other genera of Ruellieae, many of which are currently sympatric with plants of *Physacanthus* (Figures 1, S1). If a mechanism for maintenance of heteroplasmy had already been established, additional Ruellieae chloroplasts may have been thus maintained. In sum, we hypothesize ancient hybridization that established a heteroplasmic lineage and evolution of a mechanism to maintain heteroplasmy, followed by introgression between *Physacanthus* and other genera in Ruellieae.

## Conclusions

Hybridization has long been hypothesized to serve important roles in evolution [1], [48]. Numerous examples of interspecific gene exchange led to acceptance of hybridization as common in flowering plants. In contrast, natural intergeneric hybridization events, especially those involving distant relatives, are not common (but see [17]) or at least have been documented more rarely than have those between closely related species. Based on data presented in this study, the most likely scenario to explain the evolution of *Physacanthus* is one of ancient hybridization that involved phylogenetically distant lineages in the family Acanthaceae. This wide hybridization event involved not just members of different genera but members of highly divergent clades (Table 1). With the exception of incompatibilities that affect morphology or physiology, heteroplasmy is not expected to have major impact on phenotype given that most genes controlling phenotype reside in nuclear rather than organellar genomes [31]. Further, it would be difficult to detect if the parental chloroplasts were similar as indeed is expected in cases of hybridization between close relatives. Heteroplasmy may, therefore, be more common than is currently appreciated. To our knowledge, this is the first documentation of an intertribal hybridization of natural origin between evolutionarily divergent progenitors as well as the first demonstration of maintenance of heteroplasmy over substantial evolutionary time.

## Materials and Methods

### Morphological & Molecular Data

We evaluated morphological traits that have been shown in earlier studies to characterize Acantheae and Ruellieae: leaf crystals or “cystoliths,” leaf glands, filament curtains, corolla aestivation, stamen number and morphology, pollen morphology, ovule number, seed surface vestiture. Data are compiled here for the first time for *Physacanthus* and are based on study of selected herbarium specimens (Appendix S1).

We generated DNA sequence data from seven markers: one nuclear (ribosomal ITS+*5.8S*) plus six chloroplast (*trnL-trnF*, *trnT-trnL*, *trnG-trnR*, *rps16*, *trnG-trnS*, and *psbA-trnH*). We made ca. 25 attempts to generate sequence data for *Physacanthus* from another nuclear locus (*Eif3E*) for which we have data from other Acanthaceae, but without success (this result is typical of DNA extracted from herbarium specimens, as were those of all *Physacanthus*). We sampled taxa broadly across Acanthaceae [33] (Figure 1) to ensure that all lineages were represented to enable placement of *Physacanthus* with confidence. Excluding *Physacanthus*, 72 taxa were included (Appendix S2). We sampled 17 accessions representing all three species of *Physacanthus* from across its range (Guinea to the Democratic Republic of Congo; Table S1). We re-extracted DNA from three of these to test the hypothesis of heteroplasmy. Molecular data were generated and aligned following previously described protocols [32], [49]. To explore the extent of heteroplasmy, we cloned PCR products of five loci for as many *Physacanthus* accessions as possible (Table S3) plus three Acantheae and three Ruellieae using TOPO TA kits. We explored various regimes of character alignment and exclusion and, finding no difference in results, we proceeded with the approach that retained the most data [33]. Table S4 reports descriptive statistics by locus.

We conducted maximum likelihood searches in GARLI v.0.951 [50] or GARLI-Partition−0.97 beta version using a mixed model approach, with a GTR+G+I model of sequence evolution applied to the nucleotides, and a one-rate, equal-state frequencies model applied to indel characters. Bootstrap support was assessed via 100 ML replicates. Datasets were not parallel in terms of taxa and DNA accessions, and we were specifically interested in detecting differences among gene trees, thus data for each region were analyzed separately. We tested 26 alternative hypotheses regarding monophyly of *Physacanthus* via Shimodaira-Hasegawa tests (RELL optimization, 100 replicates; Table S2) implemented in PAUP* [51].

### Molecular Divergence and Time-Calibrated Phylogeny

We quantified evolutionary divergence by calculating pairwise base differences (uncorrected “p” distance in PAUP*) between Acantheae and Ruellieae and compared this to total divergence within Acanthaceae *sensu stricto*. To calculate relative molecular divergence between Acantheae and Ruellieae, we constructed a phylogram via likelihood analysis in GARLI after removing accessions of *Physacanthus* and combining data from the seven loci. This concatenated matrix consisted of 9,071 base pairs. The resulting tree was used to determine fossil constraint placements.

We estimated divergence times using the concatenated dataset. Because data were significantly non-clock-like (X^2^ = 2579.66, *p<0.0001*, df = 70), we used relaxed clock models implemented in BEAST [52]. We employed two high quality fossils to constrain the most recent common ancestor of the taxa to which the fossil could be unambiguously attributed: a macrofossil (seed) [53] and a microfossil (pollen) [54]. The seed fossil from the early to mid-Oligocene (ca. 33.7 to 28.8 mya) highly resembles seeds of extant *Acanthus* so was used to constrain *Acanthopsis*+*Acanthus* (Zero Offset = 28.8, Log St Dev = 1.1, Mean = 1.5, 5% quantile = 28.9, 95% quantile = 33.8). The pollen fossil from the upper Miocene (ca. 14.6 to 5.3 mya) highly resembles pollen of genera in the pseudocolpate clade of Ruellieae and so was used to constrain the node containing genera of that clade herein sampled (Zero Offset = 5.3, Log St Dev = 1.4, Mean = 2.5, 5% quantile = 5.4, 95% quantile = 14.7). Lognormal fossil priors, rate heterogeneity across branches modeled with an uncorrelated lognormal distribution, and a Yule process speciation tree prior were implemented. Chains were run for 20 million generations, sampling every 500th interval. Tracer v.1.5 was used to assess parameter values and verify effective sample sizes, and a maximum clade credibility tree was constructed from post burn-in trees using TreeAnnotator v.1.7.0.

### Tests for Molecular Recombination

We tested alternative hypotheses of recombination between Acantheae and Ruellieae, within Ruellieae, and within *Physacanthus* using the Recombination Analysis Tool (RAT) [55] to detect points of sequence crossover in the three gene trees in which *Physacanthus* was resolved in both Acantheae and Ruellieae (Figures 1, S1). Because RAT treats gaps as a fifth character state that could erroneously lead to identification of recombinant sites, we pruned matrix ends slightly and deleted a small number of taxa not relevant to the question (i.e, taxa in lineages other than Acantheae, Ruellieae, and *Physacanthus*). We rigorously explored all combinations of taxa of interest, including different implementations of varying sequence similarity thresholds and sliding window sizes. Alignments were manually checked to verify RAT program output.

## Supporting Information

Figure S1 **a**. *Physacanthus* is sister to the cystolith clade as well as nested in Ruellieae in ML tree based on chloroplast *psbA-trnH* dataset. Thickened branches indicate ≥70% ML bootstrap. Different accessions of *Physacanthus* are labeled as *Physacanthus batanganus*–0, *Physacanthus batanganus*–1, etc. and correspond to Table S1. Tribes Acantheae, Ruellieae, and Justicieae are labeled, as are the retinaculate clade (Acanthaceae s.s.) and cystolith clade from Figure 1. **b**. *Physacanthus* is nested within Ruellieae in ML tree based on chloroplast *trnT-trnL* dataset. Thickened branches indicate ≥70% ML bootstrap. Different accessions of *Physacanthus* are labeled as *Physacanthus batanganus*–0, *Physacanthus batanganus*–1, etc. and correspond to Table S1. Tribes Acantheae, Ruellieae, and Justicieae are labeled, as are the retinaculate clade (Acanthaceae s.s.) and cystolith clade from Figure 1. **c**. *Physacanthus* is sister to Acantheae as well as nested in Ruellieae in ML tree based on chloroplast *rps16* dataset. Thickened branches indicate ≥70% ML bootstrap. Different accessions of *Physacanthus* are labeled as *Physacanthus batanganus*–0, *Physacanthus batanganus*–1, etc. and correspond to Table S1. Tribes Acantheae, Ruellieae, and Justicieae are labeled, as are the retinaculate clade (Acanthaceae s.s.) and cystolith clade from Figure 1. **d**. *Physacanthus* is sister to Acantheae in ML tree based on chloroplast *trnG-trnS* dataset. Thickened branches indicate ≥70% ML bootstrap. Different accessions of *Physacanthus* are labeled as *Physacanthus batanganus*–0, *Physacanthus batanganus*–1, etc. and correspond to Table S1. Tribes Acantheae, Ruellieae, and Justicieae are labeled, as are the retinaculate clade (Acanthaceae s.s.) and cystolith clade from Figure 1. **e**. *Physacanthus* is sister to Acantheae in ML tree based on chloroplast *trnL-trnF* dataset. Thickened branches indicate ≥70% ML bootstrap. Different accessions of *Physacanthus* are labeled as *Physacanthus batanganus*–0, *Physacanthus batanganus*–1, etc. and correspond to Table S1. Tribes Acantheae, Ruellieae, and Justicieae are labeled, as are the retinaculate clade (Acanthaceae s.s.) and cystolith clade from Figure 1. **f**. *Physacanthus* is nested in Acantheae in ML tree based on nuclear ITS+*5.8S* dataset. Thickened branches indicate ≥70% ML bootstrap. Different accessions of *Physacanthus* are labeled as *Physacanthus batanganus*–0, *Physacanthus batanganus*–1, etc. and correspond to Table S1. Tribes Acantheae, Ruellieae, and Justicieae are labeled, as are the retinaculate clade (Acanthaceae s.s.) and cystolith clade from Figure 1.
(TIF)

Figure S2 **a**. Leaf crystals (cystoliths) are lacking in Acantheae (A) and *Physacanthus* (B) but present in Ruellieae (C–D). A. *Aphelandra dolichantha* (Acantheae; *Davidson & Donahue 8536*, RSA). B. *Physacanthus batanganus* (*Morello et al. 1261*, MO). C. *Ruellia pringlei* (Ruellieae; *Daniel 5860*, TEX). D. *Ruellia hookeriana* (Ruellieae; *Tripp & Ly 940*, RSA). cy = cystolith. **b**. Glands are present in on leaves of Acantheae (A–B), *Physacanthus* (C), and Ruellieae (D–F). A. *Stenandriopsis guineensis* (Acantheae; *Reitsma & Reitsma 705*, RSA). B. *Aphelandra dolichantha* (Acantheae; *Davidson & Donahue 8536*, RSA). C. *Physacanthus batanganus* (*Morello et al. 1261*, MO). D. *Ruellia megachlamys* (Ruellieae; *Tripp & Ly 958*, RSA). E. *Bravaisia berlandieriana* (Ruellieae; *Pitzer & Mizquez 3437*, MO). F. *Hygrophila schulli* (Ruellieae; *Jongkind & Schmidt 1727*, MO). **c**. Filament curtains are present in corollas of Ruellieae (A–B) but lacking in Acantheae and *Physacanthus*. A. *Ruellia elegans* (Ruellieae; cultivated RSABG greenhouses), longitudinal section of dorsal portion of corolla tube showing style, ovary, and filament curtain. B. *Ruellia bourgaei* (Ruellieae; cultivated RSABG greenhouses), proximal portion of corolla in foreground (distal portion in background) showing filament curtain that partitions the corolla longitudinally into two chambers. st = style, ov = ovary, fc = filament curtain. **d**. Seeds lack helically thickened trichomes in Acantheae (A–B) but have them in *Physacanthus* (C–E) and Ruellieae (F–H). A & B. *Aphelandra impressa* (Acantheae; *Tripp & Lujan 524*, RSA). C–E. *Physacanthus batanganus* (*Merello et al. 1261*, MO). F & G. *Satanocrater ruspolii* (Ruellieae; *Tripp & Ensermu 904*, RSA). H. *Ruellia humilis* (Ruellieae; *Tripp 14*, PH).
(TIF)

Figure S3 **Macromorphology of** ***Physacanthus batanganus*** **(** ***Kami 4132*** **, K; photo by M. Cheek) showing leaf variegation (A–C), inflated calyces with fused lobes (B), geniculate corolla (B), left-contort corolla aestivation (C), and rosette habit (C).**
(TIF)

Table S1 **Voucher information for accessions of** ***Physacanthus batanganus*** **(G. Braun & K. Schum.) Lindau,** ***Physacanthus cylindricus*** **C.B. Clarke, and** ***Physacanthus nematosiphon*** **(Lindau) Rendle & Britton used in this study.** * denotes re-extractions conducted to test for heteroplasmy. The 10 accessions in bold are those for which we were able to obtain sequences from more than one marker. GenBank numbers provided in Appendix S2.
(DOCX)

Table S2 **Results of SH tests of alternative phylogenetic hypotheses.** Hypotheses #1–23 reflect generic level (i.e., interspecific) tests of *Physacanthus*, whereas hypotheses #24–26 reflect intraspecific or intra-accession tests.
(DOCX)

Table S3 **The number of sequences obtained (via cloning or direct sequencing) for each of the 20 accessions of** ***Physacanthus*** **used in this study.** Entries in bold reflect the three pairs of re-extracted DNAs (*P. cylindricus*-1 is a re-extraction of *P. cylindricus*-0; *P. nematosiphon*-1 is a re-extraction of *P. nematosiphon*-0; *P. nematosiphon*-4 is a re-extraction of *P. nematosiphon*-3) –see Table S1.
(DOCX)

Table S4 **Descriptive information for data matrices used in this study.** Aligned length (including binary indel characters) refers to length after excluding ambiguous alignment sites.
(DOCX)

Text S1 **Morphological and molecular results as well as morphological discussion to support conclusions in the main text.**
(DOCX)

Appendix S1 **Selected specimens examined for morphological study of** ***Physacanthus*** **.**
(DOCX)

Appendix S2 **Voucher information and Genbank numbers (** ***trnL-trnF*** **,** ***rps16*** **,** ***trnT-trnL*** **,** ***trnG-trnS*** **,** ***trnG-trnR*** **,** ***psbA-trnH*** **, ITS+** ***5.8S*** **, -- = sequence not obtained) for all accessions used in molecular study.** Numbers in brackets refer to a series of clones for a given locus. Taxa are in phylogenetic order from out-groups through Justicieae as shown in Figure 1 except *Physacanthus* accessions listed at the end.
(DOCX)
