# Supplementary figures and images for: Origin of African Physacanthus (Acanthaceae) via Wide Hybridization
Source: PLoS One. 2013 Jan 30;8(1):e55677. doi: 10.1371/journal.pone.0055677 (PMC3559597; doi:10.1371/journal.pone.0055677)

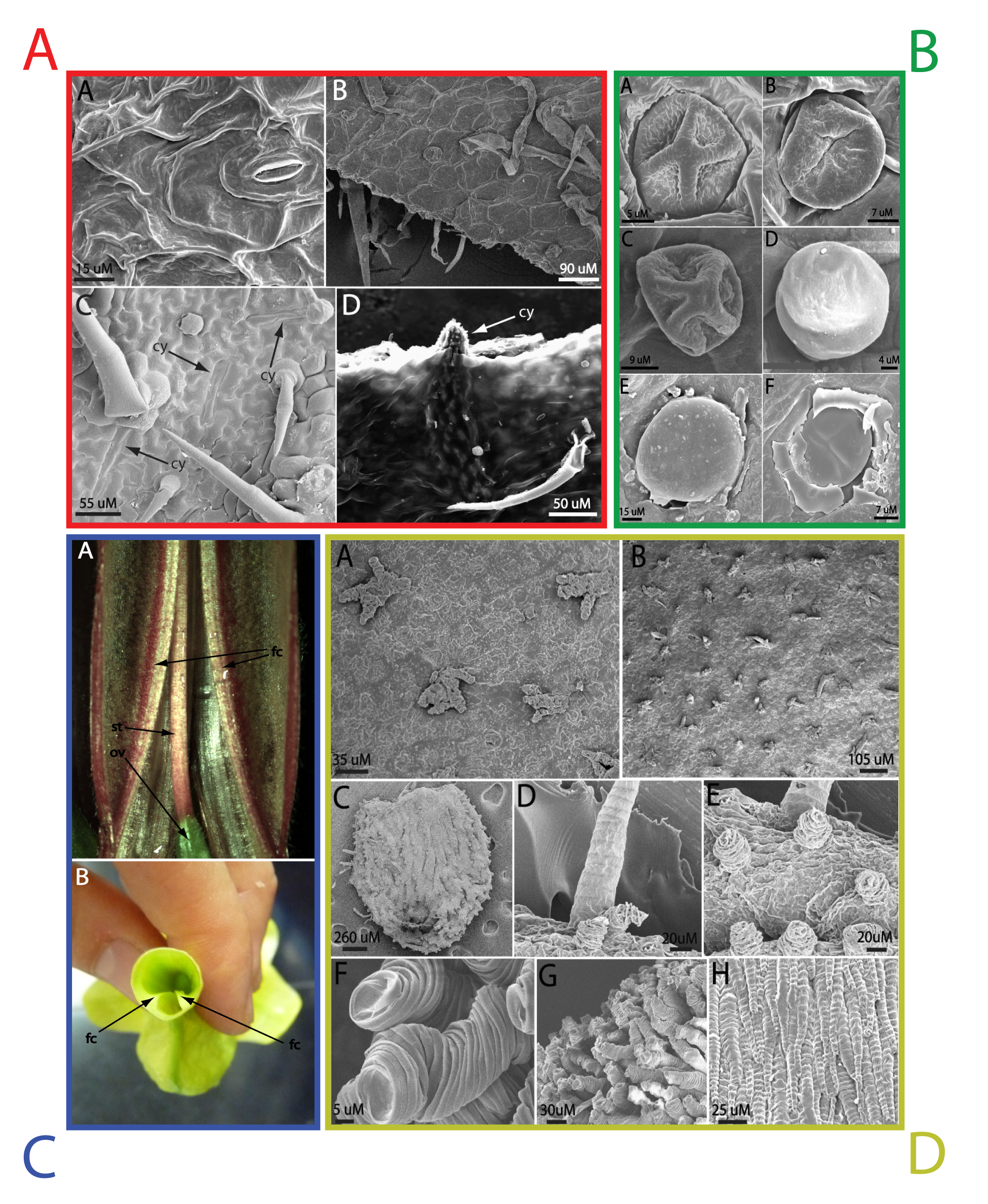

Supplement: Figure S2 — a. Leaf crystals (cystoliths) are lacking in Acantheae (A) and Physacanthus (B) but present in Ruellieae (C–D). A. Aphelandra dolichantha (Acantheae; Davidson & Donahue 8536, RSA). B. Physacanthus batanganus (Morello et al. 1261, MO). C. Ruellia pringlei (Ruellieae; Daniel 5860, TEX). D. Ruellia hookeriana (Ruellieae; Tripp & Ly 940, RSA). cy = cystolith. b. Glands are present in on leaves of Acantheae (A–B), Physacanthus (C), and Ruellieae (D–F). A. Stenandriopsis guineensis (Acantheae; Reitsma & Reitsma 705, RSA). B. Aphelandra dolichantha (Acantheae; Davidson & Donahue 8536, RSA). C. Physacanthus batanganus (Morello et al. 1261, MO). D. Ruellia megachlamys (Ruellieae; Tripp & Ly 958, RSA). E. Bravaisia berlandieriana (Ruellieae; Pitzer & Mizquez 3437, MO). F. Hygrophila schulli (Ruellieae; Jongkind & Schmidt 1727, MO). c. Filament curtains are present in corollas of Ruellieae (A–B) but lacking in Acantheae and Physacanthus. A. Ruellia elegans (Ruellieae; cultivated RSABG greenhouses), longitudinal section of dorsal portion of corolla tube showing style, ovary, and filament curtain. B. Ruellia bourgaei (Ruellieae; cultivated RSABG greenhouses), proximal portion of corolla in foreground (distal portion in background) showing filament curtain that partitions the corolla longitudinally into two chambers. st = style, ov = ovary, fc = filament curtain. d. Seeds lack helically thickened trichomes in Acantheae (A–B) but have them in Physacanthus (C–E) and Ruellieae (F–H). A & B. Aphelandra impressa (Acantheae; Tripp & Lujan 524, RSA). C–E. Physacanthus batanganus (Merello et al. 1261, MO). F & G. Satanocrater ruspolii (Ruellieae; Tripp & Ensermu 904, RSA). H. Ruellia humilis (Ruellieae; Tripp 14, PH). (TIF) [file pone.0055677.s002.tif]

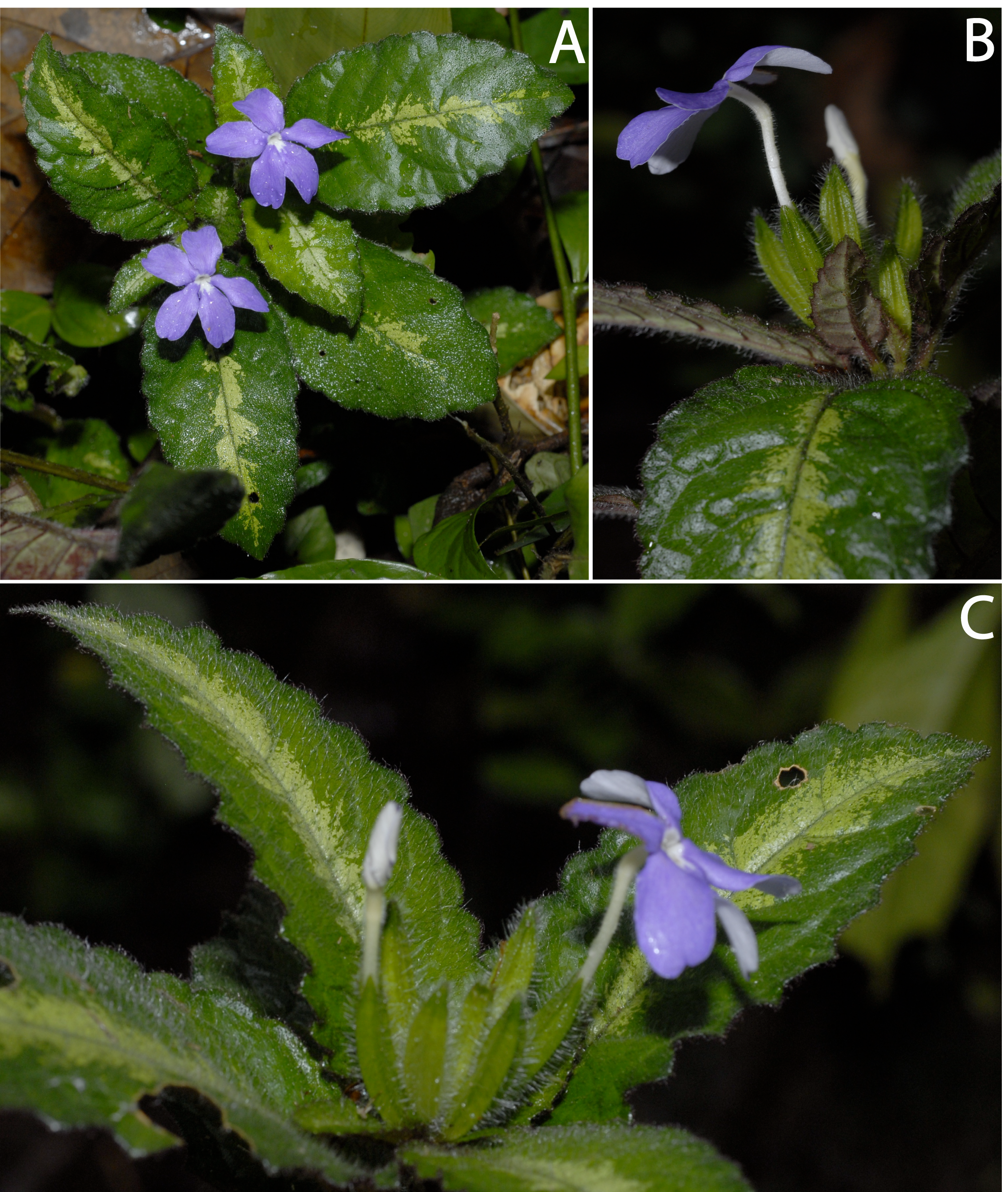

Supplement: Figure S3 — Macromorphology of Physacanthus batanganus ( Kami 4132 , K; photo by M. Cheek) showing leaf variegation (A–C), inflated calyces with fused lobes (B), geniculate corolla (B), left-contort corolla aestivation (C), and rosette habit (C). (TIF) [file pone.0055677.s003.tif]
