# Supplementary material for: Origin of African Physacanthus (Acanthaceae) via Wide Hybridization
Source: PLoS One. 2013 Jan 30;8(1):e55677. doi: 10.1371/journal.pone.0055677 (PMC3559597; doi:10.1371/journal.pone.0055677)
Supplement: Table S1 — Voucher information for accessions of Physacanthus batanganus (G. Braun & K. Schum.) Lindau, Physacanthus cylindricus C.B. Clarke, and Physacanthus nematosiphon (Lindau) Rendle & Britton used in this study. * denotes re-extractions conducted to test for heteroplasmy. The 10 accessions in bold are those for which we were able to obtain sequences from more than one marker. GenBank numbers provided in Appendix S2. (DOCX) [file pone.0055677.s004.docx]

**Table S1.**—

| Accession | Origin | Collector / Collection # | Herbarium |
| --- | --- | --- | --- |
| ***P. batanganus*-0** | Republic of Congo | *Kami 4132* | K |
| ***P. batanganus*-1** | Cameroon | *de Wilde 7780A* | MO |
| *P. batanganus*-2 | Cameroon | *Thomas 6165* | MO |
| ***P. batanganus*-3** | Eq. Guinea | *Perez-Viso 2825* | US |
| *P. batanganus*-4 | Gabon | *Arends et al. 536* | MO |
| *P. batanganus*-5 | Cameroon | *Bos 3424* | MO |
| ***P. batanganus*-6** | Gabon | *McPherson 17941* | MO |
| *P. batanganus*-7 | Gabon | *Louis 2958* | MO |
| *P. batanganus*-8 | Liberia | *Baldwin 11590* | US |
| ***P. batanganus*-9** | Gabon | *McPherson 15118* | MO |
| *P. batanganus*-10 | Gabon | *de Wilde et al. 38* | MO |
| *P. batanganus*-11 | Gabon | *Louis et al. 1400* | MO |
| ***P. cylindricus*-0** | Gabon | *de Wilde et al. 10213* | WAG |
| *P. cylindricus*-1 | Gabon | **de Wilde et al. 10213* re-extraction | WAG |
| ***P. nematosiphon*-0** | Liberia | *Jongkind 6272* | WAG |
| ***P. nematosiphon*-1** | Liberia | **Jongkind 6272* re-extraction | WAG |
| *P. nematosiphon*-2 | Liberia | *Baldwin 10241A* | US |
| *P. nematosiphon*-3 | Liberia | *Jongkind 6944* | WAG |
| ***P. nematosiphon*-4** | Liberia | **Jongkind 6944* re-extraction | WAG |
| ***P. nematosiphon*-5** | **Liberia** | ***Adam 28487*** | **MO** |
