# Supplementary material for: Origin of African Physacanthus (Acanthaceae) via Wide Hybridization
Source: PLoS One. 2013 Jan 30;8(1):e55677. doi: 10.1371/journal.pone.0055677 (PMC3559597; doi:10.1371/journal.pone.0055677)
Supplement: Table S2 — Results of SH tests of alternative phylogenetic hypotheses. Hypotheses #1–23 reflect generic level (i.e., interspecific) tests of Physacanthus, whereas hypotheses #24–26 reflect intraspecific or intra-accession tests. (DOCX) [file pone.0055677.s005.docx]

**Table S2.**—

| H# | Locus | Null | Alternative | Reject | -lnL unconstrained | -lnL constrained | Diff. | P |
| --- | --- | --- | --- | --- | --- | --- | --- | --- |
| H1 | ITS | Acantheae incl. all *Physacanthus* non-monophyletic | Acantheae (incl all *Physacanthus*) monophyletic | No | 17218.08 | 17218.70 | 0.62 | 0.47 |
| H2 | ITS | Ruellieae excl. all *Physacanthus* monophyletic | Ruellieae incl. all *Physacanthus* monophyletic | Yes | 17218.08 | 17289.82 | 71.74 | <0.05 |
| H3 | ITS | Ruellieae excl. all *Physacanthus* monophyletic | Ruellieae incl. all *Physacanthus* (and forcing backbone) monophyletic | Yes | 17218.08 | 17294.53 | 76.45 | <0.05 |
| H4 | trnLF | Ruellieae excl. all *Physacanthus* monophyletic | Ruellieae incl. all *Physacanthus* monophyletic | Yes | 9078.32 | 9140.37 | 62.05 | <0.05 |
| H5 | trnLF | Ruellieae excl. all *Physacanthus* monophyletic | Ruellieae incl. all *Physacanthus* (and forcing backbone) monophyletic | Yes | 9078.32 | 9181.90 | 103.58 | <0.05 |
| H6 | rps16 | w/in Ruellieae, *Physacanthus* non-monophyletic | w/in Ruellieae, *Physacanthus* monophyletic | Yes | 8628.04 | 8663.29 | 34.25 | <0.05 |
| H7 | rps16 | *Physacanthus* in both Ruellieae & Acantheae | *Physacanthus* monophyletic | Yes | 8628.04 | 8774.25 | 146.21 | <0.05 |
| H8 | rps16 | *Physacanthus* in both Ruellieae & Acantheae | *Physacanthus* only in Ruellieae | Yes | 8628.04 | 8662.49 | 34.45 | <0.05 |
| H9 | rps16 | *Physacanthus* in both Ruellieae & Acantheae | *Physacanthus* only in Acantheae | Yes | 8628.04 | 8976.99 | 348.95 | <0.05 |
| H10 | trnTL | w/in Ruellieae, *Physacanthus* non-monophyletic | w/in Ruellieae, *Physacanthus* monophyletic | Yes | 8306.94 | 8369.86 | 62.92 | <0.05 |
| H11 | trnTL | All *Physacanthus* are in Ruellieae | All *Physacanthus* are in Acantheae | Yes | 8306.94 | 8463.27 | 156.33 | <0.05 |
| H12 | trnTL | All *Physacanthus* are in Ruellieae | All *Physacanthus* are in Acantheae (and forcing backbone) | Yes | 8306.94 | 8526.68 | 219.74 | <0.05 |
| H13 | trnGR | w/in Ruellieae, *Physacanthus* non-monophyletic | w/in Ruellieae, *Physacanthus* monophyletic | Yes | 13566.26 | 13665.39 | 99.13 | <0.05 |
| H14 | trnGR | *Physacanthus* in both Ruellieae and Acantheae | All *Physacanthus* monophyletic | Yes | 13566.26 | 13760.14 | 193.88 | <0.05 |
| H15 | trnGR | *Physacanthus* in both Ruellieae and Acantheae | All *Physacanthus* are in Ruellieae | Yes | 13566.26 | 13690.49 | 124.23 | <0.05 |
| H16 | trnGR | *Physacanthus* in both Ruellieae and Acantheae | All *Physacanthus* are in Acantheae | Yes | 13566.26 | 13763.77 | 197.51 | <0.05 |
| H17 | trnGS | All *Physacanthus* in Acantheae | All *Physacanthus* are in Ruellieae | Yes | 13582.71 | 13919.59 | 336.88 | <0.05 |
| H18 | psbA-trnH | w/in Ruellieae, *Physacanthus* non-monophyletic | w/in Ruellieae, *Physacanthus* monophyletic | Yes | 5661.07 | 5735.03 | 73.96 | <0.05 |
| H19 | psbA-trnH | *Physacanthus* in both Ruellieae and basal to cystolith clade | All *Physacanthus* monophyletic | Yes | 5661.07 | 5757.76 | 96.69 | <0.05 |
| H20 | psbA-trnH | *Physacanthus* in both Ruellieae and basal to cystolith clade | All *Physacanthus* are in Acantheae | Yes | 5661.07 | 5763.62 | 102.55 | <0.05 |
| H21 | psbA-trnH | *Physacanthus* in both Ruellieae and basal to cystolith clade | All *Physacanthus* basal to cystolith clade | Yes | 5661.07 | 5821.34 | 160.26 | <0.05 |
| H22 | psbA-trnH | *Physacanthus* in both Ruellieae and basal to cystolith clade | All *Physacanthus* are in Ruellieae | No | 5661.07 | 5674.47 | 13.40 | 0.06 |
| H23 | psbA-trnH | *Physacanthus* in both Ruellieae and basal to cystolith clade | *Physacanthus* sister to cystolith clade are in Acantheae | No | 5661.07 | 5663.53 | 2.46 | 0.21 |
| H24 | trnGR | *P. batanganus*-6 accessions non-monophyletic | *P. batanganus*-6 accessions monophyletic | Yes | 13566.26 | 13859.23 | 292.97 | <0.05 |
| H25 | trnGR | *P. batanganus*-0 accessions non-monophyletic | *P. batanganus*-0 accessions monophyletic | Yes | 13566.26 | 13887.43 | 321.17 | <0.05 |
| H26 | trnGR | *P. cylindricus*-0 accessions non-monophyletic | *P. cylindricus*-0 accessions monophyletic | Yes | 13566.26 | 13793.34 | 227.08 | <0.05 |
