# Supplementary material for: Origin of African Physacanthus (Acanthaceae) via Wide Hybridization
Source: PLoS One. 2013 Jan 30;8(1):e55677. doi: 10.1371/journal.pone.0055677 (PMC3559597; doi:10.1371/journal.pone.0055677)
Supplement: Table S3 — The number of sequences obtained (via cloning or direct sequencing) for each of the 20 accessions of Physacanthus used in this study. Entries in bold reflect the three pairs of re-extracted DNAs (P. cylindricus-1 is a re-extraction of P. cylindricus-0; P. nematosiphon-1 is a re-extraction of P. nematosiphon-0; P. nematosiphon-4 is a re-extraction of P. nematosiphon-3) –see Table S1. (DOCX) [file pone.0055677.s006.docx]

**Table S3.**—

| Accession | ITS | *trnLF* | *trnTL* | *rps16* | *trnGR* | *trnGS* | *psbA-trnH* |
| --- | --- | --- | --- | --- | --- | --- | --- |
| *P. batanganus*-0 | - | - | - | 1 | 7 | - | 1 |
| *P. batanganus*-1 | - | - | - | 1 | 1 | 1 | - |
| *P. batanganus*-2 | - | - | - | - | 1 | - | - |
| *P. batanganus*-3 | - | 1 | - | 1 | 6 | - | - |
| *P. batanganus*-4 | - | - | - | - | 1 | - | - |
| *P. batanganus*-5 | - | - | - | - | 1 | - | - |
| *P. batanganus*-6 | 3 | 1 | - | - | 2 | 7 | - |
| *P. batanganus*-7 | - | 1 | - | - | - | - | - |
| *P. batanganus*-8 | - | - | - | - | 1 | - | - |
| *P. batanganus*-9 | - | - | 4 | 1 | 2 | - | - |
| *P. batanganus*-10 | - | - | - | - | 1 | - | - |
| *P. batanganus*-11 | - | 1 | - | - | - | - | - |
| ***P. cylindricus*-0** | **-** | **1** | **-** | **1** | **7** | **-** | **1** |
| ***P. cylindricus*-1** | **-** | **-** | **-** | **-** | **-** | **-** | **8** |
| ***P. nematosiphon*-0** | **-** | **1** | **-** | **-** | **1** | **-** | **-** |
| ***P. nematosiphon*-1** | **5** | **-** | **-** | **1** | **5** | **-** | **9** |
| *P. nematosiphon*-2 | - | - | - | - | 1 | - | - |
| ***P. nematosiphon*-3** | **-** | **1** | **-** | **-** | **-** | **-** | **-** |
| ***P. nematosiphon*-4** | **1** | **-** | **6** | **1** | **1** | **-** | **7** |
| *P. nematosiphon*-5 | 3 | 1 | - | - | 7 | - | 1 |
