# Supplementary material for: Origin of African Physacanthus (Acanthaceae) via Wide Hybridization
Source: PLoS One. 2013 Jan 30;8(1):e55677. doi: 10.1371/journal.pone.0055677 (PMC3559597; doi:10.1371/journal.pone.0055677)
Supplement: Table S4 — Descriptive information for data matrices used in this study. Aligned length (including binary indel characters) refers to length after excluding ambiguous alignment sites. (DOCX) [file pone.0055677.s007.docx]

**Table S4.**—

|  | **ITS+*5.8S*** | ***trnLF*** | ***trnTL*** | ***rps16*** | ***trnGR*** | ***trnGS*** | ***psbA-trnH*** |
| --- | --- | --- | --- | --- | --- | --- | --- |
| **Aligned Length** | 884 | 1253 | 1528 | 1139 | 1443 | 1417 | 1449 |
| **# sequences** | 92 | 79 | 114 | 77 | 148 | 120 | 64 |
| **# parsimony-informative characters** | 517 | 334 | 340 | 363 | 476 | 607 | 256 |
| **# indel characters** | 0 | 25 | 6 | 6 | 18 | 18 | 0 |
