# Supplementary material for: Origin of African Physacanthus (Acanthaceae) via Wide Hybridization
Source: PLoS One. 2013 Jan 30;8(1):e55677. doi: 10.1371/journal.pone.0055677 (PMC3559597; doi:10.1371/journal.pone.0055677)
Supplement: Text S1 — Morphological and molecular results as well as morphological discussion to support conclusions in the main text. (DOCX) [file pone.0055677.s008.docx]

Morphological and Molecular results.—

*Morphology*.—Examination of a number of macro- and micro-morphological traits indicates that species of *Physacanthus* share features with both Acantheae and Ruellieae, as shown in Figures 4, S2, S3, and described below.

*Cystoliths*.—Plants of *Physacanthus* lack cystoliths as do plants of all Acantheae that have been studied (Figure S2a). In contrast, cystoliths are present in plants of Ruellieae (Figure S2a), a lineage that is strongly supported as part of the large clade that is sister to Acantheae and that is marked by the presence of cystoliths (i.e., the cystolith clade, Figures 1, 2).

*Leaf glands*.—Sessile, patelliform glands were observed on the leaves of plants of all Ruellieae that we have studied. To our knowledge, these structures have not previously been described for other Acanthaceae. Our observation of leaves of plants of Acantheae and *Physacanthus* indicate that plants of both groups also have leaf glands (Figure S2b).

*Corolla aestivation*.—As confirmed by our study of herbarium and/or living material (or photographs of living material; Figure S3), plants of *Physacanthus* have left-contort corolla aestivation, as do those of Ruellieae. In contrast, Acantheae is characterized by plants with ascending cochlear or open aestivation (the latter occurs in genera that lack an upper corolla lip).

*Filament curtain*.—The filament curtain is a structure that partitions the corolla longitudinally into two compartments (Figure S2c); it is present in examined corollas of Ruellieae and absent from those of Acantheae. Study of flowers of *Physacanthus nematosiphon* (*Bourobou 262*, MO) and *Physacanthus* *batanganus* (*Thomas 4146*, MO), as well as Manktelow’s [56] examination of *P. nematosiphon* (*Cooper 17,* NY), revealed no discernable filament curtain.

*Stamens*.—Species of *Physacanthus* have four monothecous stamens, as do plants belonging to Acantheae (plants of Ruellieae have two to four stamens but are bithecous). Additionally, flowers of *Physacanthus* have one staminode. Staminodes characterize many genera in Ruellieae but are lacking in Acantheae. Thecae of *Physacanthus* have connective tissue with a filiform extension beyond the anther, a condition also present in Ruellieae but unknown in Acantheae. Finally, in one specimen of *Physacanthus* examined, a vestigial, second theca was seen on one of the anthers (*Hallé 2245*, K).

*Pollen*.—Species of *Physacanthus* have pollen that is tricolporate (i.e., the apertures are compound, consisting of a pore within a colpus; Figure 4) and have nine pseudocolpi. Pollen of plants of Ruellieae that we studied also have colporate (compound aperturate) pollen with nine to many pseudocolpi. In contrast, plants of Acantheae have simple aperturate, colpate pollen that lack pseudocolpi.

*Ovules*.—Plants of *Physacanthus* that we examined have 6-10 ovules per ovary, like many Ruellieae that we studied (other Ruellieae have < 6 or > 10 ovules). In contrast, four ovules per ovary is characteristic of Acantheae.

*Seeds*.—Plants of *Physacanthus* possess seeds with scattered, flexuose projections (Figure S2d). Under SEM, these appear as trichomes with helical thickenings that resemble those of Ruellieae (Figure S2d). However, the trichomes on seeds of Ruellieae are hygroscopic and become mucilaginous when wet whereas those of *Physacanthus* are not mucilaginous when wet. Seeds of most Acantheae that have been studied have some type of vestiture or sculpting on the seed surface (e.g., dendritic or strigose trichomes, pectinate scales) but lack the helical thickenings mentioned above and are not mucilaginous (except seeds of *Blepharis* which are mucilaginous [57, 58]).

Molecular Data.—DNA extracted from specimens of *Physacanthus* was recalcitrant to PCR amplification, thus our seven datasets were not parallel with regard to the presence of sequences for the 20 accessions of *Physacanthus*: Table S1 shows the number of sequences obtained for each of the 20 accessions of *Physacanthus*. Descriptive information about all matrices is provided in Table S4.

Analyses of data for the seven markers resulted in multiple phylogenetic placements for the 20 accessions of *Physacanthus*, as summarized here and detailed below. Three markers (ITS+*5.8S,* *trnL-trnF*, *trnG-trnS*) placed all *Physacanthus* sequences only among Acantheae, and one marker (*trnT-trnL*) placed all sequences for *Physacanthus* only among Ruellieae. Two markers (*trnG-trnR* and *rps16*) placed *Physacanthus* among both Acantheae and Ruellieae. One marker (*psbA-trnH*) placed some *Physacanthus* among Ruellieae and others basal to the cystolith clade.

*trnG-trnR* (Figure 2).—We obtained 45 sequences from 16 accessions of *Physacanthus* for *trnG-trnR*: 10 accessions (23 sequences) representing *P. batanganus*, one (7 sequences) representing *P. cylindricus*, and five (15 sequences) representing *P. nematosiphon*. Sequences from three accessions (two of *P. batanganus* and one of *P. cylindricus*) formed a clade (100% BS) that was sister to Acantheae (100% BS). The remaining 42 sequences were placed in three positions within Ruellieae: 40 sequences, representing all three species, were together monophyletic (100% BS) and sister to *Ruellia* (Ruellieae); one sequence of *P. batanganus* was sister to *Mimulopsis*, and another was nested among sequences for *Phaulopsis*. Neither species nor accessions of *Physacanthus* were monophyletic. Forcing monophyly of only those *Physacanthus* sequences placed with Ruellieae (H13), forcing monophyly of all *Physacanthus* (H14), and forcing all *Physacanthus* into Ruellieae (H15) or Acantheae (H16) all resulted in significantly less likely trees (*p<0.05*; Table S2). Clones from each of the other taxa (Ruellieae: *Dyschoriste*, *Hygrophila*, *Phaulopsis*; Acantheae: *Blepharis*, *Crossandra*, *Stenandrium*) were monophyletic (in the case of *Phaulopsis* inclusive of the sequence of *P. nematosiphon* just mentioned), and each of these clades received bootstrap support (>70%).

*psbA-trnH* (Figure S1a).—We obtained data from five accessions of *Physacanthus* for *psbA-trnH*: one accession (one sequence) representing *P. batanganus*, one (nine sequences) representing *P. cylindricus*, and three (17 sequences) representing *P. nematosiphon*. Fifteen of the 27 sequences were placed in Ruellieae, although the lineage, as well as several basal branches, is only weakly supported. The single sequence of *P. batanganus* was sister to *Ruellia* with weak support. The other 14 form a strongly supported clade that also included *Dyschoriste* and was sister to *Phaulopsis* with strong support. Neither species nor accessions were monophyletic in this larger clade. The remaining 12 formed a clade (86% BS) that was sister to the entire cystolith clade and composed of two homogeneous subclades: four *P. cylindricus* sister to eight *P. nematosiphon*, with strong support for each subclade. Forcing monophyly of the 15 sequences placed in Ruellieae resulted in a significantly less likely tree (H18, *p<0.05*; Table S2). Forcing monophyly of all 27 accessions (H19), all 27 into Acantheae (H20), or all basal to the cystolith clade (H21) resulted in significantly less likely trees (*p<0.05*; Table S2). In contrast, forcing all 27 into Ruellieae (H22, *p=0.06*), or forcing those basal to the cystolith clade into Acantheae (H23, *p=0.21*) resulted in trees that could not be rejected with these data (Table S2).

*trnT-trnL* (Figure S1b).—We obtained data from four clones of *Physacanthus batanganus* (one specimen) plus six clones of *Physacanthus nematosiphon* (one specimen) for *trnT-trnL*. All *Physacanthus* sequences were nested within Ruellieae (100% BS), and alternative hypotheses of their placement in Acantheae, with (H11) or without (H12) enforcing the Acanthaceae backbone, resulted in significantly less likely trees (*p<0.05*; Table S2). All but two of the *Physacanthus* sequences formed a clade (100% BS) that was sister to a clade comprising all sequences of *Phaulopsis*. Two *Physacanthus* sequences (one from each species) were together sister to *Mimulopsis* (99% BS). As such, neither *Physacanthus* as a whole nor the sequences from either of the two species were monophyletic, and forcing their monophyly resulted in a significantly less likely tree (H10, *p<0.05*; Table S2). Clones from each of five other taxa (Ruellieae: *Dyschoriste*, *Hygrophila*, *Phaulopsis*; Acantheae: *Blepharis*, *Crossandra*,) formed clades; in contrast, one of seven cloned sequences of *Stenandrium pilosulum* was placed several nodes away from the others with all intervening branches strongly supported.

*rps16* (Figure S1c).—We obtained one sequence from each of seven accessions of *Physacanthus* for *rps16*: four representing *P. batanganus*, one representing *P. cylindricus*, and two representing *P. nematosiphon*. *Physacanthus batanganus*-1, *P. cylindricus*-0, and *P. nematosiphon*-1 were resolved in Ruellieae (100% BS), the first sister to *Mimulopsis* and the other two together sister to *Dyschoriste* with strong support for these placements. The other four accessions formed a clade (100% BS) sister to Acantheae (100% BS), consisting of three accessions of *P. batanganus* and one of *P. nematosiphon*. Forcing monophyly of the three *Physacanthus* in Ruellieae could not be rejected by these data (H6, *p=0.14*), but forcing the monophyly of all seven *Physacanthus* (H7), or forcing all into Ruellieae (H8) or Acantheae (H9) resulted in significantly less likely trees (*p<0.05*; Table S2).

*trnG-trnS* (Figure S1d).—We obtained eight sequences from two accessions of only one species, *P. batanganus*, for *trnG-trnS.* All eight formed a clade (100% BS) that was sister to Acantheae (100% BS). Forcing all eight *Physacanthus* sequences into Ruellieae (H17) resulted in significantly worse trees (*p<0.05*; Table S2). Clones from all other taxa (Ruellieae: *Dyschoriste*, *Hygrophila*, *Phaulopsis*; Acantheae: *Blepharis*, *Crossandra*, *Stenandrium*) were monophyletic, and each of these was supported by bootstrap (>70%).

*trnL-trnF* (Figure S1e).—We obtained one sequence from each of eight accessions of *Physacanthus* for *trnL-trnF*: four representing *P. batanganus*, one of *P. cylindricus*, and three of *P. nematosiphon*. All sequences formed a clade (100% BS) that was sister to Acantheae (100% BS). Sequences of *P. batanganus* were monophyletic (71% BS) whereas *P. cylindricus* was resolved within the clade containing *P. nematosiphon*, albeit without support. Alternative hypotheses of the placement of all *Physacanthus* in Ruellieae, both with and without enforcement of the Acanthaceae backbone, were significantly less likely (H4 & H5, *p<0.05*; Table S2).

*ITS + 5.8S* (Figure S1f).—We obtained data from four accessions of *Physacanthus* for ITS+*5.8S*: one accession (three sequences) representing *P. batanganus* and three (nine sequences) representing *P. nematosiphon* (Table S3). All accessions of *Physacanthus* formed a monophyletic but weakly supported (i.e., < 70% bootstrap (BS)) group. Within *Physacanthus*, sequences from *P. batanganus* (all from accession 6) were monophyletic (95% BS), but those representing *P. nematosiphon* were not owing to a clone from accession 6 that was sister to all other sequences of *Physacanthus*. Further, the two accessions of *P. nematosiphon* for which multiple sequences were obtained (accessions 1 and 7) were not monophyletic although branch lengths between them are very short indicating that these sequences are very similar. The *Physacanthus* clade was sister to *Crossandra*, albeit with weak support. Acantheae were not monophyletic in this analysis (contrast Figure S1f and Figure 2) but our data cannot reject monophyly of Acantheae (including all *Physacanthus*) (H1, Hypothesis 1, *p=0.47*; Table S2). Alternative scenarios for placement of *Physacanthus* in Ruellieae, whether or not the backbone of relationships among lineages of Acanthaceae was enforced (i.e., as depicted in Figure 2), can be rejected by these data (H2 & H3, *p<0.05*; Table S2). All but one clone from each of the other taxa (i.e., *Dyschoriste*, *Hygrophila*, *Phaulopsis*: Ruellieae; *Crossandra*, *Stenandrium*: Acantheae) formed clades, each with ML bootstrap support (> 70% BS); one clone of *Crossandra* was placed several nodes away from the others but with weak support for intervening branches.

In sum, three loci supported a monophyletic genus *Physacanthus* (ITS+*5.8S*, *trnL-trnF*, *trnG-trnS*, the latter with sequences from only one of three species). Of nine accessions (four of *P. batanganus*, two of *P. cylindricus*, three of *P. nematosiphon*) for which we obtained more than one sequence for any given locus, only one, *P. batanganus*-6 (ITS+*5.8S* & *trnG-trnS* data) was monophyletic. In marked contrast, for the other six taxa of Acanthaceae for which we had sequences from multiple clones, in all but three of 23 cases (i.e., species by genic region combinations), the conspecific sequences formed strongly supported clades exclusive of other sequences. The exceptions were: (1) the clade that included all *trnG-trnR* clones of *Phaulopsis imbricata* also included one clone of *Physacanthus batanganus*-9 (Figure 2); (2) one of five cloned ITS+*5.8S* sequences of *Crossandra greenstockii* was placed several nodes away from the other clones although all intervening branches were not supported (Figure S1f); (3) one *trnT-trnL* clone of *Stenandrium pilosulum* was placed several strongly supported nodes away from the others (Figure S1b). In no cases were clones of these other genera (i.e., *Phaulopsis*, *Crossandra*, and *Stenandrium*) placed in entirely different major clades of Acanthaceae.

*SH Tests*.—As described above and in Table S4, 23 of the 26 alternative hypotheses (i.e., all except H1, H22, and H23) were rejected by the relevant data. Notably, ML searches implementing the three constraints that forced monophyly of sequences from the same accession when these were not monophyletic in the ML results (*trnG-trnR*: H24-26) all resulted in significantly worse trees (*p<0.05*; Table S2).

Morphological Discussion.—

In the context of placing *Physacanthus* within Acanthaceae s.l., it is important to note that plants of *Physacanthus* have fruits with retinacula, strongly supporting placement within Acanthaceae s.s. (Figure 2). Our survey of other morphological traits of *Physacanthus* further confirms that these plants share features with both Acantheae and Ruellieae, as discussed below (Figures 2, S2, and S3). First, however, two of the traits that we studied did not lend insight into more precise placement of *Physacanthus* within Acanthaceae s.s.:

(1) Although leaf glands have previously been reported from Ruellieae [59, 60] but not Acantheae, we documented their presence on leaves of both *Physacanthus* and Acantheae (Figure S2b). This trait merits synoptic study across Acanthaceae. We suspect that presence of sessile glands on leaves is widespread and thus likely symplesiomorphic in the family and uninformative with respect to the relationship of *Physacanthus* to other genera.

(2) Seed surfaces have not been comprehensively surveyed across Acanthaceae. Under SEM, the remarkable trichomes with helical thickenings on seeds of *Physacanthus* most closely resemble those of Ruellieae, but the former are not mucilaginous like those of Ruellieae. As described above, seeds of Acantheae have diverse surfaces although to our knowledge none have the remarkable helical thickenings that characterize Ruellieae and *Physacanthus* (Figure S2d) except those of *Blepharis*, which, also like Ruellieae, become mucilaginous when wet. *Blepharis* is nested distally in Acantheae and thus its seed features are almost certainly homoplasious with those of seeds of Ruellieae. Certainly, seed surface vestiture and ‘behavior’ merit synoptic study in Acanthaceae. If the trichomes with helical thickenings on seeds of *Physacanthus* are synapomorphic with those of Ruellieae or *Blepharis*, then loss of the mucilaginous response in *Physacanthus* must be postulated.

The other traits that we studied are potentially phylogenetically informative. Like Acantheae, plants of *Physacanthus* have four monothecous stamens and lack a filament curtain as well as leaf cystoliths. Like Ruellieae, plants of *Physacanthus* have left-contort corolla aestivation, compound aperturate pollen, and ovaries with > four ovules.

The traits that plants of *Physacanthus* share with Acantheae are mostly symplesiomorphic for all Acanthaceae: absence of leaf cystoliths (Figure S2a), absence of a filament curtain (Figure S2c), and four stamens. Indeed, these are likely symplesiomorphic for a phylogenetically much more inclusive group within Lamiales. In contrast, monothecous stamens are no doubt apomorphic. So far as is known, all Acantheae have four monothecous stamens. A few other taxa of Acanthaceae have monothecous anthers but only two stamens (e.g., *Hypoestes*, Justicieae; 61). However, these genera are all phylogenetically distant from Acantheae and nested distally in large clades of plants with dithecous anthers such that monothecous anthers have clearly evolved homoplasiously. The combination of four stamens with monothecous anthers is, so far as we know, unique to Acantheae and *Physacanthus*. However, occasional observation of a second, vestigial theca or anthers with connective tissue extending beyond the theca / thecae in *Physacanthus* adds complexity to this otherwise synapomorphic combination of staminal features for *Physacanthus* and Acantheae. Dithecous stamens and connective tissue extensions are traits characteristic of Ruellieae.

The three traits that link *Physacanthus* to Ruellieae (compound aperturate pollen, >4 ovules/ovary, left-contort corolla aestivation) also occur in other lineages of the cystolith clade (Figure 2). First, whereas only pollen with simple apertures is known from Acantheae (where it is symplesiomorphic), the cystolith clade as a whole is marked by pollen with compound apertures (Figure 2). Additionally, pollen of Acantheae lack pseudocolpi, whereas that of most genera of Ruellieae and Justicieae have pseudocolpi (Figure 2), which we here propose as a synapomorphy for Ruellieae + Justicieae. Pollen traits thus link *Physacanthus* to the larger cystolith clade (compound apertures) and, within that clade, to Ruellieae + Justicieae (pseudocolpi). Pollen morphology has historically been, and continues to be, an extremely important character to delimit taxa in Acanthaceae [35, 36, 62].

Second, McDade et al. [33] proposed reduction to four ovules as a synapomorphy for Acanthaceae s.l. except Nelsonioideae. Like some Ruellieae, Andrographideae are also marked by a secondary increase to six or more ovules per ovary [33]; as these groups are not closely related, this trait is homoplasious. In contrast, Acantheae almost invariably have four ovules per ovary and exceptions involve reduction rather than increase in ovule number. As our results do not otherwise yield evidence of a relationship between *Physacanthus* and Andrographideae, ovule number is consistent with placement of *Physacanthus* in Ruellieae.

Third, so far as known, among Acanthaceae s.s. only *Lankesteria* and *Whitfieldia* of Whitfieldieae share left-contort aestivation with *Physacanthus* (Figure 2 and Figure S3) and Ruellieae [63]. The trait is also present among some members of clades of Acanthaceae s.l. that are basal to the retinaculate clade (i.e., several Thunbergioideae and *Avicennia* [64]. In the absence of other data to support a relationship of *Physacanthus* to Whitfieldieae or the basalmost members of Acanthaceae s.l., contort aestivation is consistent with placement of *Physacanthus* in Ruellieae.

In sum, there are fixed, distinctive morphological features of *Physacanthus* that suggest placement sister to the cystolith clade of Acanthaceae s.s. (e.g., absence of cystoliths) or a relationship to Acantheae (i.e., four monothecous anthers). Conflicting with this pattern are traits that argue instead for placement of *Physacanthus* in the cystolith clade (i.e., compound aperturate pollen), with Ruellieae + Justicieae (i.e., pseudocolpate pollen), or with Ruellieae (i.e., ovule number, corolla aestivation).

Supporting references.—

56. Manktelow M (2000) The filament curtain: a structure important to systematics and pollination biology in the Acanthaceae. Bot J Linn Soc 133: 129-160.

57. Gutterman Y, Witztum A (1977) The movement of integumentary hairs in *Blepharis* *ciliaris* (L.) Burtt. Bot Gaz 138: 29-34.

58. Vollesen K (2000) Blepharis (Acanthaceae): a taxonomic revision. London: Royal Botanic Gardens, Kew.

59. McDade LA, Tripp EA (2007) A synopsis of Costa Rican *Ruellia* (Acanthaceae), with descriptions of four new species. Brittonia 59: 199-216.

60. Tripp EA (2007) Evolutionary relationships within the species-rich genus *Ruellia* (Acanthaceae). Syst Bot 32: 628-649.

61. Darbyshire I, Vollesen K (2007) The transfer of the genus *Peristrophe* to *Dicliptera* (Acanthaceae) with a new species described from Eastern Africa. Kew Bull 62: 119-128.

62. Daniel TF (1998) Pollen morphology of Mexican Acanthaceae: diversity and systematic significance. Proc Cal Acad Sci 50: 217:256.

63. Manktelow M, McDade LA, Oxelman B, Furness CA, Balkwill M-J (2001) The enigmatic tribe Whitfieldieae (Acanthaceae): delimitation and phylogenetic relationships based on molecular and morphological data. Syst Bot 26: 104-119.

64. Schönenberger J (1999) Floral structure, development and diversity in *Thunbergia* (Acanthaceae). Bot J Linn Soc 130: 1-36.
