# Supplementary material for: Origin of African Physacanthus (Acanthaceae) via Wide Hybridization
Source: PLoS One. 2013 Jan 30;8(1):e55677. doi: 10.1371/journal.pone.0055677 (PMC3559597; doi:10.1371/journal.pone.0055677)
Supplement: Appendix S1 — Selected specimens examined for morphological study of Physacanthus . (DOCX) [file pone.0055677.s009.docx]

**Supporting Appendix S1.—**

*Physacanthus batanganus*: *Baldwin 6088* (K, MO); *P. batanganus*: *Enti 42641* (K, MO, P); *P. batanganus*: *Merello et al. 1261* (K, MO); *P. batanganus*: *Bos 6032* (MO, WAG); *P. batanganus*: *Louis & Nzabi 2958* (MO, WAG); *P. batanganus*: *Reitsma et al.* 1074 (NY, WAG); *P. batanganus*: *Wilks 3721* (MO, WAG); *P. batanganus*: *Wilks 2568* (MO, WAG); *P. batanganus*: *Arends et al. 536* (MO, WAG); *P. batanganus*: *Thomas & Mambo 4229* (K, MO, WAG); *P. batanganus*: *de Wilde et al. 475* (MO, WAG); *P. batanganus*: *Bourobou 262* (MO, WAG); *P. batanganus*: *Reitsma* *& Reitsma* *2769* (NY, WAG); *P. batanganus*: *Louis et al. 1400* (MO, WAG); *Physacanthus cylindricus: Breteler 12963* (WAG); *P. cylindricus: Hallé 2188* (P); *P. cylindricus: Mann 1669 pro parte* (K); *P. cylindricus: Nguema Miyono 1376* (WAG); *P. cylindricus: Sanford 5911* (K); *Physacanthus nematosiphon*: *Adam 21471* (K, P); *P. nematosiphon*: *Chevalier 19670* (K, P); *P. nematosiphon*: *Jacques-Félix 960* (P); *P. nematosiphon*: *Jongkind 6944* (WAG); *P. nematosiphon*: *Enti & Hall GC39141* (K).
