# Supplementary material for: Origin of African Physacanthus (Acanthaceae) via Wide Hybridization
Source: PLoS One. 2013 Jan 30;8(1):e55677. doi: 10.1371/journal.pone.0055677 (PMC3559597; doi:10.1371/journal.pone.0055677)
Supplement: Appendix S2 — Voucher information and Genbank numbers ( trnL-trnF , rps16 , trnT-trnL , trnG-trnS , trnG-trnR , psbA-trnH , ITS+ 5.8S , -- = sequence not obtained) for all accessions used in molecular study. Numbers in brackets refer to a series of clones for a given locus. Taxa are in phylogenetic order from out-groups through Justicieae as shown in Figure 1 except Physacanthus accessions listed at the end. (DOCX) [file pone.0055677.s010.docx]

**Supporting Appendix S2.—**

**Outgroups:** *Martynia annua* L. – AF067065, EU529040, EU529107, EU528979, KC118389, --, AF169854, Mexico, *Jenkins 97-149* (ARIZ); *Sesamum indicum* L. – AF067067, EU529060, EU529125, EU528998, JQ781019, EU531713, AF169853, cultivated, Mexico, *Jenkins 97-141* (ARIZ). **Nelsonioideae:** *Elytraria imbricata* (Vahl) Pers. – AF061819, EU529020, EU529086, EU528957, KC118367, --, AF169852, Arizona, USA, *McDade & Jenkins 1155* (ARIZ); *Nelsonia canescens* (Lam.) Spreng. – AF363668, EU529047, EU529114, EU528985, --, --, JQ691822, Panama, *Daniel et al. 5254* (CAS); *Staurogyne letestuana* Benoist – KC420626, EU529061, EU529126, EU528999, JQ781020, KC420678, JQ691801, cultivated, Belgium, *NBG-B 200000119-77* (BR). ***Avicennia* lineage:** *Avicennia bicolor* Standl. – AY008818, EU529007, EU529072, EU528943, JQ780995, KC420633, EU528877, Costa Rica, *Borg 10* (S); *Avicennia germinans* (L.) Stearn – AY008819, EU529008, EU529073, EU528944, KC118339, KC420634, EU528878, cultivated, University of Connecticut greenhouses; *Avicennia marina* (Forssk.) Vierh. – JQ728990, EU529009, EU529074, EU528945, KC118340, JX448688, EU528879, Australia, *Schwartzbach 97-A1* (KE). **Thunbergioideae:** *Mendoncia cowanii* (S. Moore) Benoist – EU528935, EU529042, EU529109, EU528981, --, --, EU528899, Madagascar, *Hearn Mad-3* (PH); *Mendoncia glabra* (Poepp. & Endl.) Nees – KC420613, EU529043, EU529110, EU528982, EU528982, KC420647, --, Bolivia, *Daniel & Wood 10176* (CAS); *Mendoncia phytocrenoides* Benoist – AF167330, EU529044, EU529111, EU528983, JQ781005, --, AF169849, Cameroon, *Schönenberger 50* (K); *Thunbergia alata* Bojer ex Sims – AF061820, EU529063, EU529130, --, --, --, AF169850, cultivated, from commercial seed (ARIZ); *Thunbergia coccinea* Wall. – --, EU529064, EU529131, EU529000, --, --, EU528909, cultivated, Zurich Botanical Garden, *Schönenberger 144* (Z); *Thunbergia erecta* (Benth.) T. Anderson – AF061821, EU529065, EU529132, EU529001, KC118454, --, AF169851, cultivated, Missouri Botanical Garden, *Missouri 802421* (MO). **Acantheae:** *Acanthus sennii* Chiov. – DQ059148, DQ054856, DQ059224, EU528941, KC118335, KC420629, DQ028415, Ethiopia, *Friis et al. 7006* (C); *Acanthopsis disperma* Nees – KC420610, DQ059142, KC118457, DQ059218, --, KC420628, DQ028411, South Africa, *Balkwill et al. 11780* (J); *Aphelandra boyacensis* Leonard – AF061828, DQ059196, EU529070, DQ059275, KC118337 KC118338, KC420631, AF169759, Colombia, *McDade 989* (DUKE); *Aphelandra leonardii* McDade – AF063112, DQ059205, EU529071, DQ059287, KC118338, KC420632, AF169761, Panama, *McDade 310* (DUKE); *Blepharis maderaspatensis* (L.) B. Heyna ex Roth – DQ059160, DQ059160, [EU529077, KC118458, KC118459, KC118460, KC118461, KC118462], [DQ059237, KC420570, KC420571, KC420572, KC420573, KC420574], DQ059238, [KC118342, KC118343, KC118344, KC118345, KC118346, KC118347, KC118348] --, DQ028423, South Africa, *McDade 1292* (PH); *Blepharis subvolubilis* C.B. Clarke – DQ054864, DQ059164, --, DQ059241, KC118349, --, --, South Africa, *Balkwill et al. 10850* (J); *Crossandra greenstockii* S. Moore – DQ054871, DQ059171, [DQ054871, KC118463, KC118464, KC118465], [DQ059250, KC420575, KC420576, KC420577, KC420578, KC420579, KC420580, KC420581], [KC118352, KC118353, KC118354, KC118355, KC118356, KC118357, KC118358, KC118359, KC118360], KC420638, [DQ028427, KC420538, KC420539, KC420540, KC420541], South Africa, *McDade & Balkwill 1241* (J); *Geissomeria longiflora* Lindl. – DQ054904, DQ059211, KC118475, DQ059293, KC118369, KC420639, DQ028465, Brazil, *Wasshausen 2337* (US); *Sclerochiton harveyanus* Nees – DQ054866, DQ059167, KC118506, DQ059244, KC118446, --, DQ028424, South Africa, *Balkwill 12274* (J); *Stenandriopsis guineensis* (Nees) Benoist – DQ054877, DQ059179, EU529127, DQ059258, JQ781021, KC420679, DQ028434, cultivated, Royal Botanic Gardens Kew, *K1990-2299* (K); *Stenandrium pilosulum* (S.F. Blake) T.F. Daniel – AF061827, DQ059191, [EU529128, KC118507, KC118508, KC118509, KC118510, KC118511, KC118512, KC118513], [DQ059270, KC420606, KC420607, KC420608, KC420609], [KC118447, KC118448, KC118449, KC118450, KC118451, KC118452, KC118453], --, [AF169758, KC420567, KC420568, KC420569], Mexico, *Van Devender & Reina 97-434* (ARIZ). **Barlerieae:** *Acanthostelma thymifolium* (Chiov.) Bidgood & Brummitt – EU528912, EU529004, EU529068, EU528939, KC118334, --, EU528874, Somalia, *Thulin et al. 10650* (UPS); *Acanthura mattogrossensis* Lindau – EU528913, EU529005, EU529069, EU528940, --, --, EU528875, Bolivia, *Bruderreck 173* (K); *Barleria lupulina* Lindl. – AF289758, EU529010, EU529075, EU528946, JQ780996, --, AF169751, cultivated, San Francisco Conservatory of Flowers, *Daniel s.n.* (CAS); *Barleria repens* Nees – EU528915, EU529011, EU529076, EU528947, KC118341, --, AF169750, cultivated, Missouri Botanical Garden, *Missouri 97003* (MO); *Crabbea acaulis* N.E. Br. – EU528918, EU529016, EU529082, EU528953, JQ781000, --, EU528885, South Africa, *Balkwill et al. 11649* (J); *Golaea migiurtina* Chiov. – EU528921, EU529022, EU529088, EU528959, KC118370, --, EU528888, Somalia, *Thulin et al. 10665* (UPS); *Lasiocladus villosus* Benoist – EU528927, EU529032, EU529098, EU528969, KC118383, --, EU528892, Madagascar, *Daniel et al. 10427* (CAS); *Lepidagathis alopecuroidea* (Vahl) R. Br. Ex Griseb. – AF167702, EU529033, --, EU528971, KC118384, --, AF169753, Panama, *Daniel et al. 8066* (CAS); *Lepidagathis falcata* Nees – EU528929, --, EU529100, EU528972, --, --, EU528894, Thailand, *Suddee 957* (K); *Lepidagathis formosensis* C.B. Clarke ex Hayata– EU528930, EU529034, EU529101, EU528973, KC118385, --, EU528895, Taiwan, *Bartholomew et al. 7654* (CAS); *Lepidagathis scabra* C.B. Clarke– EU528931, EU529035, EU529102, EU528974, KC118386, --, EU528896, South Africa, *McDade & Balkwill 1238* (J); *Lophostachys chiapensis* Acosta Cast. – EU528932, EU529036, EU529103, EU528975, KC118387, --, EU528897, Mexico, *Daniel 8374* (CAS); *Lophostachys pubiflora* Lindau – EU528933, EU529037, EU529104, EU528976, --, --, --, Bolivia, *Daniel et al. 10106* (CAS); *Lophostachys uxpanapensis* Acosta Cast. – EU528934, EU529038, EU529105, EU528977, KC118388, --, EU528898, Mexico, *Garcia 539* (ARIZ). **Andrographideae:** *Andrographis paniculata* (Burm. f.) Nees – EU528914, EU529006, --, EU528942, --, JQ922119, --, cultivated, *McDade 1329* (RSA-POM); *Cystacanthus turgida* G. Nicholson – EU528919, EU529017, EU529083, EU528954, JQ781001, --, EU528886, cultivated, Royal Botanic Gardens Kew, *1996-479* (K); *Gymnostachyum ceylanicum* Arn. & Nees – EU528922, EU529023, EU529089, EU528960, --, --, --, cultivated, San Francisco Conservatory of Flowers, *Daniel s.n.* (CAS); *Indoneesiella echioides* (L.) Sreem. – EU528923, EU529026, EU529092, EU528963, KC118378, --, KC420551, cultivated, San Francisco Conservatory of Flowers, *Daniel s.n.* (CAS); *Phlogacanthus thyrsiflorus* Nees – EU528938, EU529053, EU529120, EU528993, KC118401, --, EU528907, India, *Lindburg 200* (DAV). **Whitfieldieae:** *Camarotea souiensis* Scott-Elliot – EU528916, EU529013, EU529079, EU528949, JQ780998, KC420635, EU528881, Madagascar, *Decary s.n.* (US); *Chlamydacanthus dichrostachyus* Mildbr. – KC420611, KC420527, EU529080, EU528950, KC118351, KC420636, EU528882, Tanzania, *Semsei 804* (BR); *Chlamydacathus euphorbioides* Lindau – EU528917, EU529014, EU529081, EU528951, JQ780999, --, EU528883, Madagascar, *Daniel et al. 10445* (CAS); *Chlamydacanthus lindavianus* H. Winkl. – AF194435, EU529015, --, EU528952, --, KC420637, EU528884, Tanzania, *Borhidi et al. 85464* (UPS); *Forcipella* sp. Baill. – EU528920, EU529021, EU529087, EU528958, KC118368, --, EU528887, Madagascar, *Daniel et al. 10432* (CAS); *Lankesteria brevior* C.B. Clarke – AF194436, KC420529, EU529095, EU528966, KC118380, KC420642, --, Ghana, *Manktelow et al. 117* (UPS); *Lankesteria elegans* T. Anderson – EU528925, EU529030, EU529096, EU528967, KC118381, KC420643, EU528890, Cameroon, *Etuge & Thomas 466* (CAS); *Lankesteria glandulosa* Benoist – EU528926, EU529031, EU529097, EU528968, KC118382, KC420644, EU528891, Madagascar, *Daniel et al. 10435* (CAS); *Leandriella oblonga* Benoist – EU528928, --, EU529099, EU528970, --, KC420645, EU528893, Madagascar, *DuPuy et al. MB767* (P); *Whitfieldia elongata* (P. Beauv.) De Wild. & T. Durand – AF195518, EU529066, KC118514, EU529002, KC118455, --, EU528910, cultivated, Uppsala Botanical Garden, *Manktelow 682* (UPS). ***Neuracanthus* lineage:** *Neuracanthus africanus* T. Anderson ex S. Moore – EU528936, EU529048, EU529115, EU528986, KC118390, KC420648, EU528900, South Africa, *McDade et al. 1258* (J); *Neuracanthus ovalifolius* (Fiori) Bidgood & Brummitt – EU528937, EU529049, EU529116, EU528988, KC118391, KC420649, EU528902, Somalia, *Friis et al. 5032* (K); *Neuracanthus umbraticus* Bidgood & Brummitt – KC420615, EU529051, EU529118, EU528991, KC118392, JQ781006, EU528905, cultivated, San Francisco Conservatory of Flowers, *Daniel 6770.5* (CAS). **Ruellieae:** *Acanthopale confertiflora* (Lindau) C.B. Clarke – --, KC420526, KC118456, JQ781022, JQ7801022, EF214651, JQ781035, EF214470, Madagascar, *Phillipson 2117* (MO), *Dyschoriste albiflora* Lindau – KC420612, KC420528, [KC118466, KC118467, KC118468, KC118469, KC118470, KC118471, KC118472, KC118473, KC118474], [GQ995605, KC420582, KC420583, KC420584, KC420585, KC420586], [EF214606, KC118361, KC118362, KC118363, KC118364, KC118365, KC118366], GQ995666, [EF214416 KC420543, KC420544], Zambia, *Luwiika et al. 580* (MO); *Hygrophila corymbosa* Lindau – AF063120, EU529024, [EU529090, KC118476, KC118477, KC118478, KC118479, KC118480, KC118481, KC118482, KC118483, KC118484], [EU528961, KC420587, KC420588, KC420589], [KC118371, KC118372, KC118373, KC118374, KC118375, KC118376, KC118377], KC420640, [AF169836, KC420545, KC420546, KC420547, KC420548, KC420549, KC420550], cultivated, Missouri Botanical Garden, *MO897223* (MO); *Mimulopsis solmsii* Schweinf. – KC420614, --, KC118485, --, EF214616, --, EF214427, Uganda, *ATBP 530* (MO); *Pararuellia alata* H.P. Tsui – KC420616, KC420530, KC118486, --, KC118394, KC420650, --, China, *Zhiduan 960432* (MO); *Phaulopsis imbricata* Sweet. – KC420617, KC420531, [KC118487, KC118488, KC118489, KC118490, KC118491, KC118492, KC118493, KC118494], [JX444034, KC420590, KC420591, KC420592, KC420593, KC420594, KC420595, KC420596, KC420597], [EF214619, KC118395, KC118396, KC118397, KC118398, KC1183959, KC118400], KC420651, [EF214430, KC420552, KC420553, KC420554], Tanzania, *Bidgood et al. 4589* (MO); *Ruellia humilis* Nutt – AF482604, AF482538, KC118505, EU431038, EF214678, GQ995632, EF214508, Pennsylvania, *Tripp 14* (PH); *Sanchezia speciosa* Leonard – AF063113, EU529059, EU529124, EU528997, EU431005, --, AF169835, cultivated, Duke University Greenhouses, *McDade 1180* (ARIZ); *Strobilanthes dyeriana* Mast. – --, EU529062, EU529129, JX444046, JX443973, JX443892, EU528908, cultivated, *McDade 1328* (RSA-POM). **Justiceae:** *Anisacanthus thurberi* (Torr.) A. Gray – AF063122, EU087483, EU081113, EU081046, KC118336, KC420630, AF169846, Arizona, USA, *Jenkins 05-007* (ARIZ) [rps16, trnTL, trnGS, trnGR, psbA-trnH] and Arizona, USA, *Van Devender 88-150* (ARIZ) [trnLF, ITS]; *Dicliptera extenta* S. Moore – AF289724, EU529018, EU529084, EU528955, --, --, KC420541, cultivated, private garden, South Africa, *McDade 1306* (J); *Ecbolium syringifolium* (Vahl) Vollesen – AF289743, EU087529, DQ372435, DQ372480, --, AF289786, Madagascar, *Daniel & Butterwick 6733* (CAS); *Justicia adhatoda* L. – AF289734, DQ059214, EU081176, EU081109, KC118379, LC420641, AF289773, cultivated, University of Arizona Campus Arboretum, *Barr 60-393* (ARIZ); *Mackaya bella* Harv. – AF289751, EU529039, EU529106, EU528979, JQ781003, KC420646, AF289796, cultivated, Strybing Arboretum, San Francisco, USA, *Daniel s.n.* (CAS); *Metarungia galpinii* (Baden) Baden – AF289737, EU529046, EU529113, EU528984, --, --, AF289776, South Africa, *Daniel 9323* (CAS); *Odontonema tubaeforme* Kuntze – AF063127, DQ059215, DQ372462, DQ059297, KC118393, --, AF169748, cultivated, Duke University Greenhouses, *McDade 1182* (ARIZ); *Ptyssiglottis pubisepala* (Lindau) B. Hansen – AF289744, EU529055, DQ372438, DQ372483, KC118416, --, AF289787, Papua New Guinea, *Daniel 6630* (CAS); *Rhinacanthus gracilis* Klotzsch – AF289727, EU529057, EU529122, EU528995, JQ781009, KC420677, AF289766, cultivated, San Francisco Conservatory of Flowers, *Daniel s.n.* (CAS). ***Physacanthus*.** **“*P. batanganus-*0”** *Physacanthus batanganus* (J. Braun & K. Schum.) Lindau – --, KC420532, --, --, [KC118402, KC118403, KC118404, KC118405, KC118406, KC118407, KC118408], --, Republic of Congo, *Kami 4131* (K); **“*P. batanganus-*1”** *Physacanthus batanganus* (J. Braun & K. Schum.) Lindau – --, KC420533, --, KC420598, --, --, --, Cameroon, *de Wilde 7780A* (MO); **“*P. batanganus-*2”** *Physacanthus batanganus* (J. Braun & K. Schum.) Lindau – --, --, --, --, KC118418, --, --, Cameroon, *Thomas 6165* (MO); **“*P. batanganus-*3”***Physacanthus batanganus* (J. Braun & K. Schum.) Lindau – KC420618, --, --, --, [KC118419, KC118420, KC118421, KC118422, KC118423, KC118424], --, --, Equatorial Guinea, *Perez Viso 2825* (US); **“*P. batanganus-*4”** *Physacanthus batanganus* (J. Braun & K. Schum.) Lindau – --, --, --, --, KC118425, --, --, Gabon, *Arends et al. 36* (MO); **“*P. batanganus-*5”** *Physacanthus batanganus* (J. Braun & K. Schum.) Lindau – --, --, --, --, KC118426, --, --, Cameroon, *Bos 3424* (MO); **“*P. batanganus-*6”** *Physacanthus batanganus* (J. Braun & K. Schum.) Lindau – KC420619, --, --, [KC420599, KC420600, KC420601, KC420602, KC420603, KC420604, KC402605], [KC118427, KC118428] KC118429 --, [KC420555, KC420556, KC420557], Gabon, *McPherson 17941* (MO); **“*P. batanganus-*7”** *Physacanthus batanganus* (J. Braun & K. Schum.) Lindau – KC420620, --, --, --, --, --, --, Gabon, *Louis 2958* (MO); **“*P. batanganus-*8”** *Physacanthus batanganus* (J. Braun & K. Schum.) Lindau – --, --, --, --, KC118417, --, --, Liberia, *Baldwin 11590* (US); **“*P. batanganus-*9”** *Physacanthus batanganus* (J. Braun & K. Schum.) Lindau – --, [KC420534, KC420535], [KC118495, KC118496, KC118497, KC118498], --, [KC118444, KC118445], --, --, Gabon, *McPherson 15118* (MO); **“*P. batanganus-*10”** *Physacanthus batanganus* (J. Braun & K. Schum.) Lindau – --, --, --, --, KC118430, --, --, Gabon, *de Wilde et al. 38* (MO); **“*P. batanganus-*11”** *Physacanthus batanganus* (J. Braun & K. Schum.) Lindau – KC420621, --, --, --, --, --, --, Gabon, *Louis et al. 1400* (MO); **“*P. cylindricus-*0”** *Physacanthus cylindricus* C.B. Clarke – KC420622, KC420536, --, --, [KC118409, KC118410, KC118411, KC118412, KC118413, KC118414] KC118415, KC420652, --, Gabon, *de Wilde 10213* (WAG); **“*P. cylindricus-*1”** *Physacanthus cylindricus* C.B. Clarke – --, --, --, --, --, psbA-trnH [KC420653, KC420654, KC420655, KC420656, KC420657, KC420658, KC420659, KC420660], --, Gabon, *de Wilde 10213* (WAG); **“*P. nematosiphon-*0”** *Physacanthus nematosiphon* (Lindau) Rendle & Britten – KC420623, --, --, --, KC118415, --, --, Liberia, *Jongkind 6272* (WAG) **“*P. nematosiphon-*1”** *Physacanthus nematosiphon* (Lindau) Rendle & Britten – --, --, --, --, [KC118431, KC118432, KC118433, KC118434, KC118435], [KC420661, KC420662, KC420663, KC420664, KC420665, KC420666, KC420667, KC420668, KC420669], [KC420558, KC420559, KC420560, KC420561, KC420562], Liberia, *Jongkind 6272* (WAG); **“*P. nematosiphon-*2”** *Physacanthus nematosiphon* (Lindau) Rendle & Britten – --, --, --, --, KC118429, --, --, Liberia, *Baldwin 10241A* (US); **“*P. nematosiphon-*3”** *Physacanthus nematosiphon* (Lindau) Rendle & Britten – KC420624, --, --, --, --, --, --, Liberia, *Jongkind 6944* (WAG); **“*P. nematosiphon-*4”** *Physacanthus nematosiphon* (Lindau) Rendle & Britten – --, KC420537, [KC118499, KC118500, KC118501, KC118502, KC118503, KC118504], --, KC118436, [KC420670, KC420671, KC420672, KC420673, KC420674, KC420675, KC420676], KC420563, Liberia, *Jongkind 6944* (WAG); **“*P. nematosiphon-*5”** *Physacanthus nematosiphon* (Lindau) Rendle & Britten – KC420625, --, --, --, [KC118437, KC118438, KC118439, KC118440, KC118440, KC118441, KC118442, KC118443], --, [KC420564, KC420565, KC420566], Liberia, *Adam 28487* (MO);
